# Design and Development of ‘Building Opportunities for Optimal physical activity Skills Training in children with Down Syndrome (BOOST‐DS)’ Programme and its Content Validation through Consensus using Modified Delphi method

**DOI:** 10.1111/jir.70119

**Published:** 2026-05-22

**Authors:** V. Megha Jain, Bhamini K. Rao, Selvam Ramachandran, Delphi Expert Panel, B. S. Santhosh Kanna, Deepalaxmi Paresh Poojari, Deepa Metgud, Kirti Joshi, Mithula Vijaykumar, Radhika Attal, Rajitha Alva, Sanjay Tejraj Parmar, Shreekanth D. Karnad, Smitha Elizabeth Joseph, Tanochni Mohanty

**Affiliations:** ^1^ Department of Physiotherapy Manipal College of Health Professions, Manipal Academy of Higher Education, Manipal Udupi Karnataka India

**Keywords:** agility, balance, children with Down syndrome, coordination, endurance, exercise programme, flexibility, physical activity participation, strength

## Abstract

**Background:**

Children with Down syndrome (DS) often do not meet daily physical activity requirements, which affects their participation in daily activities and overall quality of life. Structured physical activity programmes could address these issues, but accessible, participation‐based activities are limited.

**Method:**

The Building Opportunities for Optimal physical activity Skills Training in children with Down Syndrome (BOOST‐DS) programme was designed, developed and content validated in three phases. A conceptual framework was developed from the relevant literature, followed by the identification physical activity components through brainstorming and discussions. The final content validation phase involved expert consensus using a modified Delphi method.

**Results:**

The BOOST‐DS identified 62 active play items across six domains of physical activity. After three Delphi rounds, 53 items achieved expert agreement.

**Conclusion:**

The content‐validated BOOST‐DS programme includes 53 play‐based activities across six physical activity domains to enhance participation outcomes in children with DS.

**Trial Registration:** The study was registered in the Clinical Trial Registry of India: CTRI/2023/08/056291 on 5 August 2023

## 1 Introduction

Children with Down syndrome (DS) often have brain structural variations that contribute to physical and intellectual impairments affecting their fundamental motor skills and learning and memory (Jain et al. 2021). Concurrently, a high risk of chronic comorbidities, including early‐onset Alzheimer's disease, obesity, diabetes, hypothyroidism and cardiovascular diseases, fosters increased sedentary behaviour and obesity, which in turn exacerbates the very comorbidities and functional limitations that restrict activity and negatively impact quality of life (QoL) (Alqahtani et al. 2024; Di Fabrizio et al. 2021; El and Hüzmeli 2020; Pitetti et al. 2013). This trend continues as children with DS age, leading to reduced physical activity and fitness beyond childhood, negatively impact their ability to perform activities of daily living, executive function, community participation and overall QoL (Alhammad et al. 2024; Alhusaini et al. 2020; Phillips and Holland 2011).

A robust body of scientific evidence has demonstrated the beneficial effects of physical activity interventions in children with DS. Physical activity interventions ranging from neuromuscular and treadmill training to innovative exergaming have been shown to improve strength, mobility and balance functions in children with DS (Bahiraei et al. 2023; Kamińska et al. 2023; Rodríguez‐Grande et al. 2022). Physical activity can even stimulate cognitive functions, potentially improving cognitive or brain reserve and mental health for children with intellectual disabilities (Yang et al. 2022), and is effective in enhancing attention, working memory and executive functions (Mero Piedra et al. 2024; Wang et al. 2023; Zhu et al. 2023).

The benefits are clear, yet a critical gap remains in how these physical activity interventions are delivered. Several studies emphasise the need for structured, accessible physical activity programmes that increase social interaction and motivation for children with DS (Wentz et al. 2021; Downs et al. 2013; Barr and Shields 2011). However, many existing physical activity programmes for children with DS are confined to clinical or home settings that focus on individualised training (Alba‐Rueda et al. 2022; Hojlo et al. 2022; Regaieg et al. 2020), thereby limiting crucial opportunities for social interaction and peer engagement. Recognising this significant oversight, experts in the field have issued a call to action—to shift from a clinical model to one that emphasises the delivery of physical activity interventions in a school‐based setting, where children with DS can practice physical skills alongside their peers, building real‐world competence and confidence (Johnson et al. 2021; Wentz et al. 2021; Shields and Synnot 2016). Schools are not just centres for academic learning; they are natural hubs for social and motor development, offering a structured, inclusive and equitable space to integrate physical activity into a child's daily routine (Shields and Synnot 2016).

In India, DS ranks among the most prevalent chromosomal conditions, with an estimated incidence varying regionally between 1.2 and 1.4 per 1000 live births, accounting for approximately 37 000 new cases annually (Kaur and Kaur 2020; Lakhan and Kishore 2016). In the Indian context, children with DS are offered educational services through special school settings. Despite the strong evidence on the benefits of physical activity programmes for health and QoL in children with DS, in India, there is a lack of context‐specific, structured programmes. In response to this emergent need, this study was conceptualised to build opportunities for physical activity skills training for children with DS in special school settings, integrating the best global practices with region‐specific insights.

## 2 Method

The investigators aimed to contextualise the design, development and content validation of ‘Building Opportunities for Optimal physical activity Skills Training in children with Down Syndrome (BOOST‐DS)’, a play‐oriented and structured participation‐based physical activity p rogramme for children with DS to be implemented in special school settings with minimal resources. The study adopted a pragmatic research paradigm, which prioritises practical solutions and real‐world applicability, and is well‐suited for intervention development and content validation. The study employed a mixed‐methods design, as it allows the integration of both qualitative (brainstorming, expert panel feedback) and quantitative (consensus ratings) approaches to achieve the most meaningful and actionable outcomes. The study was implemented in three phases. The objective of the first phase was to systematically review the literature around the relevant theories and principles to design a participation‐based physical activity programme for children with DS. The second phase focused on identifying the domains of physical activity interventions and developing a list of contextualised play‐oriented participation‐based physical activity interventions. In the third and final phases, the investigators blended global evidence and local expertise, contextualising the BOOST‐DS, with the goal of driving its feasibility, utility and sustainability. This phased approach ensures that the programme is evidence‐based, culturally attuned and practical to implement in Indian special schools. The investigators obtained the necessary approvals from the Institutional Research Committee and the Ethics Committee before commencing the study. The study participants, including postgraduate trainees and paediatric physiotherapy experts, provided written informed consent to participate in the study. The data collected during the development phase from the trainee participants and during the validation phase from the paediatric physiotherapy experts were anonymized. Data were stored digitally and securely on a password‐protected computer system accessible only to the research team. The study findings are reported according to the ACCORD (Accurate Consensus Reporting Document) checklist provided in Supporting Information S1.

### 2.1 Design of the BOOST‐DS Programme

Reviewing the empirical evidence from the broader literature, the investigators contemplated designing the BOOST‐DS programme with a focus on the ‘participation’ construct as its central tenet. The participation construct is recognised to meaningfully engage the children with DS in physical activity, which positively influences the cognitive, social and emotional domains in addition to physical well‐being. The investigators identified and adopted SAAFE physical education teaching principles—supportive, active, autonomous, fair and enjoyable (Lubans et al. 2017)—as an umbrella concept in designing the BOOST‐DS programme and integrated it with interrelated themes of participation constructs—preference, attendance and involvement, activity competence and sense of self—described by Imms et al. (2016). By combining SAAFE principles with these participation elements, the BOOST‐DS was designed to be evidence‐informed, culturally appropriate and provide equitable opportunities, ensuring that physical activity sessions are inclusive and engaging for children with DS and sustainable for implementation in Indian special schools. Table 1 lists the design features of the BOOST‐DS programme.

**TABLE 1:** Design features of the BOOST‐DS program.

Theories and principles	Determinants of participation			Dimensions of participation		
SAAFE >	Support		Autonomy	Active	Fair	Enjoyable
SDT >	Competence	Relatedness	Autonomy	—	—	—
Participation constructs >	Activity competence	Sense of Self	Preference	Attendance	Involvement	
Variables of BOOST‐DS programme	Challenging yet safe opportunities Provide specific feedback Positive reinforcement	Social context Facilitative language, behaviours and expectations	Option choice (item selection) Action choice (difficulty level selection)	Activity circuit Active warm up Reduced transition time Gradient progression in activity levels Avoiding elimination	Competition—focus progression on mastery level (personal progression) and NOT performance level Equity Adaptive rules Use of Adaptive equipment	Variety support—variety and variability in activities Novelty Use of music Activity NOT as a punishment

*Note:* The colour in the table matches with the colour used in the Conceptual Model of the BOOST‐DS programme presented in Figure 1.

**FIGURE 1:**
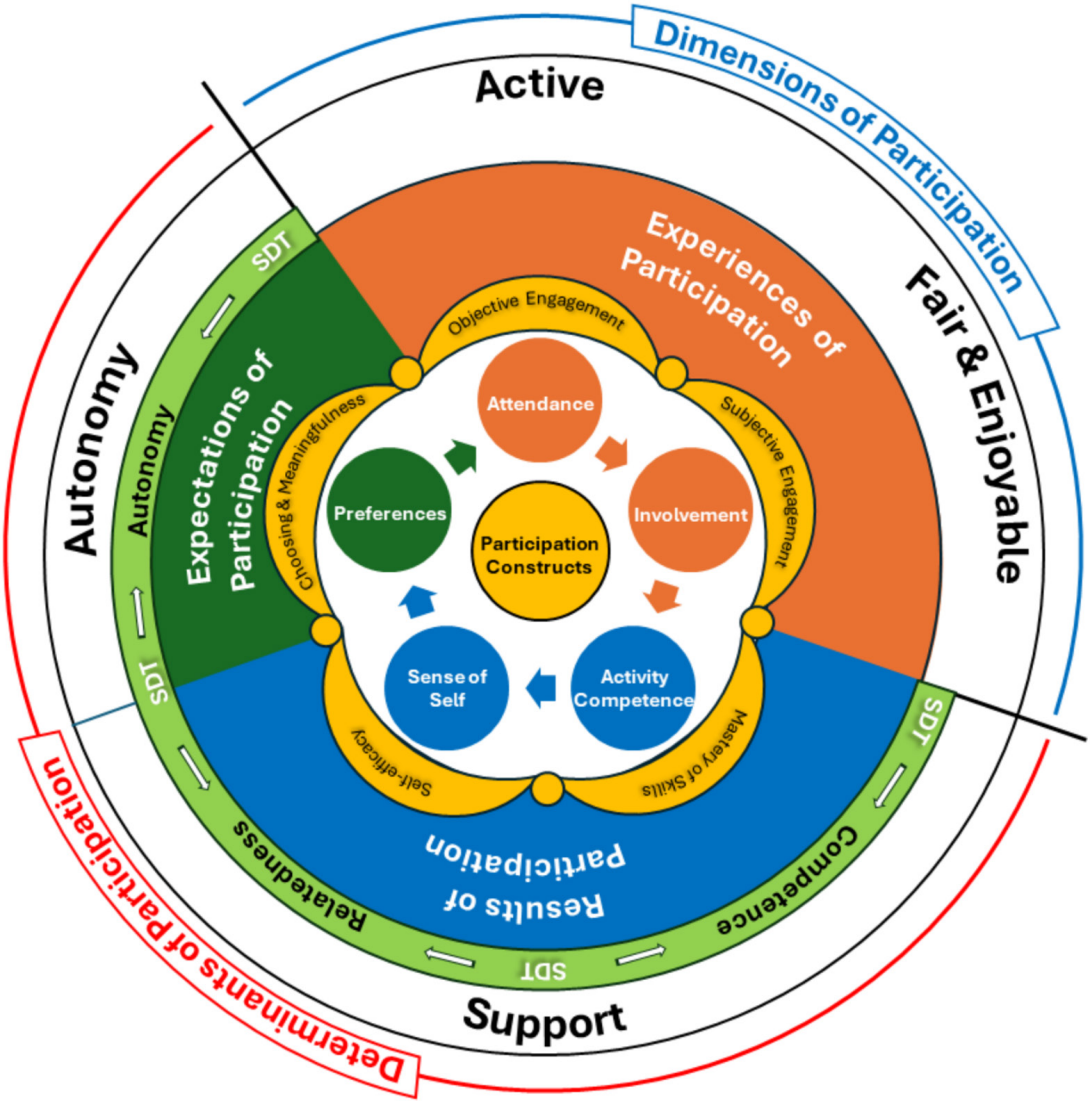
Conceptual model of the BOOST‐DS programme.

The ‘Support’ principle of the SAAFE framework driven by self‐determination theory fosters intrinsic motivation for engaging in physical activity by fulfilling the psychological needs of autonomy (making personal choices in activities), competence (achieving satisfaction in challenging tasks) and relatedness (feeling socially connected with peers regardless of abilities) (Lubans et al. 2017; Teixeira et al. 2012). The ‘Active’ principle ensures that sessions are highly engaging, progressively challenging and minimise idle time, which aligns with ACSM's physical activity recommendations for children with intellectual disabilities—combining aerobic (40%–80% VO2 max), resistance (60%–80% of 1RM) and flexibility activities for 30–60 min per session. The ‘Autonomous’ principle offers opportunities for children to make meaningful choices in activity selection (option choice) and difficulty levels (action choice), which are personally valuable and enjoyable, driving intrinsic motivation. The ‘Fair’ principle focuses on providing equitable opportunities in a competitive format aimed at personal mastery rather than performance success. Finally, the ‘Enjoyable’ principle ensures participation by designing physical activities to be fun and varied, using games and music, and promoting positive emotions and strengthening social bonds while avoiding punitive approaches. Thus, our conceptual framework has incorporated the evidence‐informed elements of intrinsic motivation and engagement into physical activity participation in children with DS in a school setting. Figure 1 presents a conceptual model linking the interrelated concepts of SAAFE principles and participation constructs to inform the design of the BOOST‐DS programme. With the identified design features, the investigators set out to develop the BOOST‐DS programme for children with DS, obtained the necessary institutional approvals and registered the study with the Clinical Trial Registry.

### 2.2 Development of the BOOST‐DS Programme

Grounding on the proposed conceptual framework, the investigators contemplated the ‘BOOST‐DS’ to be organised, structured, accessible, to incorporate motivating and engaging play activities, to implement them in a special school setting and to improve overall participation outcomes in children with DS. During the development phase, the investigators conducted a systematic search of specific databases and identified eight studies that met the search criteria. The search strategy details are provided in the Supporting Information S2. These studies highlighted six main domains of physical activity—strength, endurance, flexibility, balance, coordination and agility. However, only a few studies have reported play‐based interventions in school settings that could be implemented with minimal resources. Previous work included a feasibility study, *Go2Play*, designed to build fundamental motor and social skills in children with intellectual disabilities using simple infrastructure (McGarty et al. 2021), a quasi‐experimental study showing that games improved gross motor skills (Priyono et al. 2021) and another study noting gains in basic motor skills through games (Astuti et al. 2024). None of these studies have described active play or game content in detail. This gap motivated the investigators to identify culturally relevant, play‐based physical activity items for children with DS in India.

To generate ideas, the lead investigator applied the rapid ideation brainstorming approach (Ritter and Mostert 2018) involving four postgraduate trainees in paediatric physiotherapy with experience facilitating play‐based activities for children with DS. The process began with each trainee recalling games and sports from school and community experiences during their childhood, then making ‘random connections’ to adapt those activities for children with DS. For example, the classic hopscotch game was adapted by drawing large, colourful shapes on the floor rather than using numbers. The rule is not just to hop on one foot but to jump to a specific shape or colour called out by a peer. Thus, the focus shifts from purely motor planning to include cognitive processing of shapes and colours, all while using the familiar structure of the hopscotch game. This ‘random connection’ technique bypasses conventional, linear thinking and fosters creative ideas and solutions.

In the final step, the trainees, as a group, reflected and iterated on their ideas of active play items. This process yielded 83 active play items mapped to six domains of physical activity, which were then reviewed for suitability using four criteria—task complexity, cognitive demand, equipment requirements and safety. The 83 active play items identified through the rapid ideation brainstorming approach are listed in the Supporting Information S3. Following group discussion and consultation with the lead investigator, 21 active play items were delisted that were too complex, cognitively demanding, equipment‐heavy or unsafe. The final set of 62 active play items was then subjected to the validation phase of the study.

### 2.3 Content Validation of the BOOST‐DS Programme

During this phase, the investigators used a modified Delphi approach to reach expert consensus on the 62 play items identified across six domains of physical activity. The Delphi approach was modified by initiating the process of consensus with predetermined activity items derived from prior rapid ideation sessions and a literature review, rather than open‐ended questions. The approach was also modified to include a synchronous face‐to‐face online meeting with moderated discussion, after three asynchronous iterative rounds of surveying to analyse preliminary results and reach consensus, rather than maintaining anonymity throughout the study. The process was coordinated by the lead investigator, who had more than a decade of experience running physical activity programmes for children with DS in special school settings. A purposive sampling strategy was employed to select participants with the requisite expertise to ensure they possess specialised knowledge and practical experience necessary to provide informed judgements during the validation process. A panel of paediatric physiotherapy experts, comprising academics, clinicians and researchers with at least 2 years of professional experience was selected. Care was taken to ensure diversity in both expertise and geographic representation. Invitations explaining the Delphi process and requesting consent to participate in the study were sent via email and followed up with phone calls. Among the 15 experts who met the inclusion criteria, 11 agreed to participate in all three Delphi rounds, falling within the recommended panel size of 8–23 experts for Delphi consensus studies (Shang 2023). The panel members were blinded to one another and completed all three sequential rounds of the consensus process asynchronously via an online questionnaire developed in JotForm. The structured validation questionnaire to invite expert responses is provided in the Supporting Information S4. The experts rated each active play item on a 5‐point Likert scale for relevance, appropriateness, clarity of instructions and feasibility and provided written feedback or suggestions. The experts were given 15 days to respond for the first two rounds (Rounds 1 and 2) and 7 days for the last round (Round 3), with reminder emails and phone calls sent when necessary.

**FIGURE 2:**
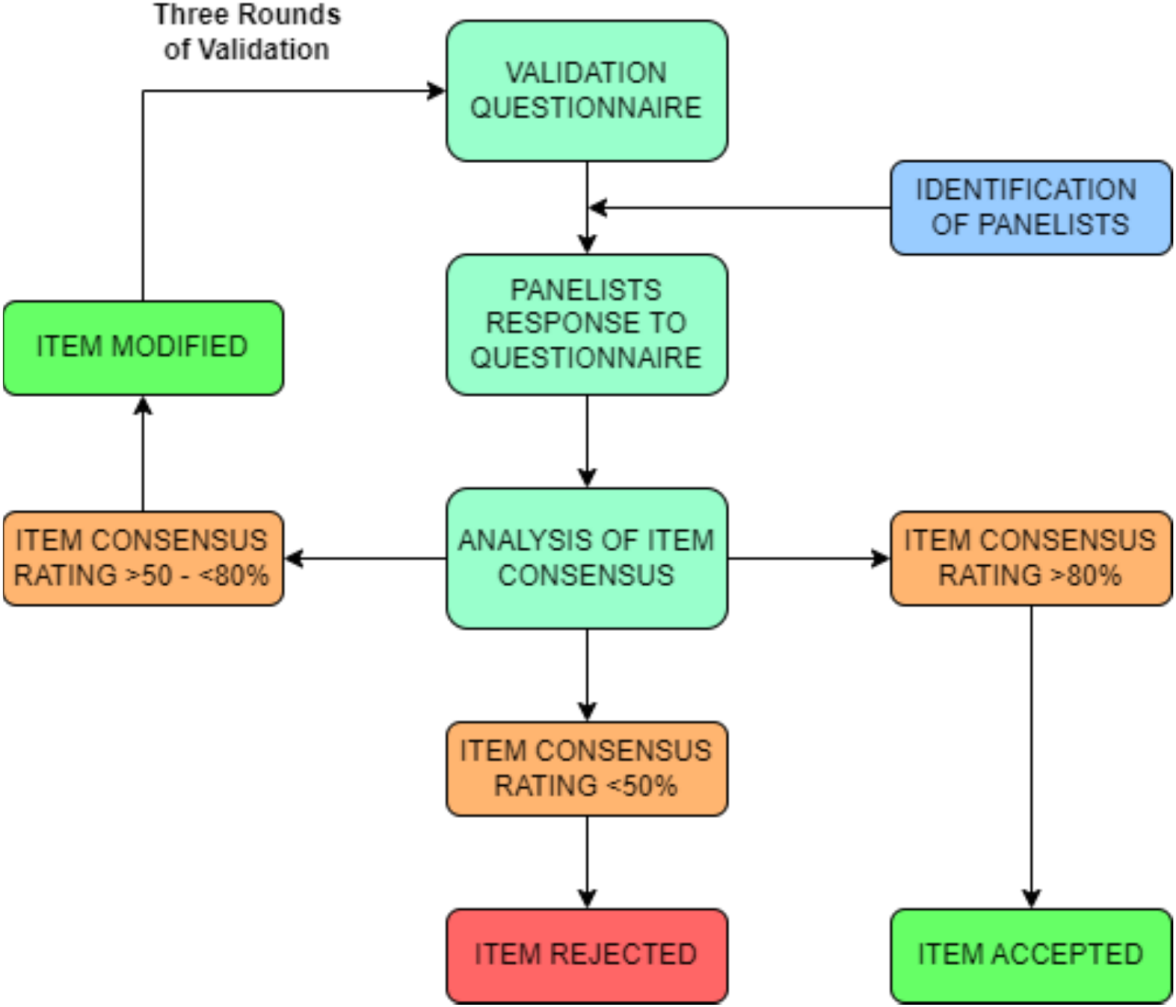
Content validation through consensus process using modified Delphi method.

The investigators used multiple strategies to ensure rigour in the consensus‐building process. The credibility of the responses was ensured through sustained engagement with the experts via email and phone, and through member checking of summarised Delphi feedback with panellists. The consensus on activity item selection was exclusively on expert feedback, bracketing researcher assumptions during analysis to limit selection bias. The investigators maintained a clear and detailed audit trail, documenting all the responses of consensus rounds, modifications and acceptance decisions. A predefined threshold (≥ 80% agreement on Likert ratings of 4 or 5) was established to determine consensus for each activity item, ensuring transparency in decision‐making. Items that achieved at least 80% agreement (score ≥ 4) in each category were accepted. Items with 50%–79% agreement were revised and re‐evaluated in the next round, while those with less than 50% agreement were removed (Shang 2023). Three Delphi rounds were conducted during these periods: Round 1 (30 November 2023 to 21 January 2024), Round 2 (1 February to 16 March 2024) and Round 3 (18–23 March 2024). After each round, the investigators reviewed the expert ratings and feedback to decide whether to retain, modify or delete an item. Once all rounds were completed, the item content validity index (I‐CVI) for each item was calculated via Microsoft Excel. The summary of expert responses to the content validation questionnaire is provided in the Supporting Information S5. The content validation process through consensus is illustrated in Figure 2.

The content‐validated BOOST‐DS programme offers a variety of choices for facilitators to promote physical activity in children with DS. Furthermore, the active play items identified in the programme involve simple instructions that children with DS could follow. During the implementation of the BOOST‐DS programme, the children with DS can be grouped based on their intellectual functioning and adaptive skills. The appropriate choice of active play—option choice and difficulty choice—can accommodate the varying levels of motor development and adaptive abilities in children with DS.

## 3 Results

Table 2 presents the demographic characteristics of the panel of experts who participated in the study, and Table 3 summarises the consensus process. During the first consensus round, 62 items were validated by the expert panel. Of the 62 items, 24 items received consensus (> 80% agreement), and 38 items did not receive consensus (< 80% and > 50% agreement). The investigators rejected no items during this round. The items that achieved consensus in each domain in the first round were strength (3), endurance (6), coordination (2), balance (5), agility (6) and flexibility (2). The items that did not receive consensus were modified based on expert feedback. During the second round of consensus, 38 items were validated. Of the 38 items, 23 received consensus (> 80% agreement), 9 were rejected (< 50% agreement) and 6 did not reach consensus (> 50% and < 80% agreement). The items that achieved consensus in each domain in the second round were strength (2), endurance (5), coordination (4), balance (5), agility (5) and flexibility (2). All items under the flexibility (4) domain received consensus at the end of the second Round. The six items that did not reach a consensus were further modified. Six items were validated during the third round of consensus, and all received consensus (> 80% agreement). The items that achieved consensus in each domain in the third round were strength (1), endurance (1), coordination (1), balance (2) and agility (1). The investigators, at the end of three rounds of Delphi consensus, accepted a total of 53 items across the strength (6), endurance (12), coordination (7), balance (12), agility (12) and flexibility (4) domains and rejected a total of 9 items across the strength (4), endurance (2), coordination (1) and agility (2) domains.

**TABLE 2:** Demographic details of the panellists.

Demographic characteristics		*n* = 11
Gender	Male	3
	Female	8
Highest qualification	Doctoral	5
	Postgraduate	5
	Graduate	1
Experience	> 2 to < 6 years	1
	> 6 to < 10 years	2
	> 10 years	8
Designation	Professor	1
	Associate professor	1
	Assistant professor	5
	PhD scholar	1
	Clinical physiotherapist	1
	Head of department	2
Geographic location of panellists	Bangalore, Karnataka, India	4
	Belagavi, Karnataka, India	1
	Dharwad, Karnataka, India	1
	Mangalore, Karnataka, India	1
	Manipal, Karnataka, India	1
	Chennai, Tamil Nadu, India	2
	Los Angeles, California, USA	1

**TABLE 3:** Summary of the consensus process for item content validation.

Domains (‘*n*’ items)	Round 1			Round 2			Round 3		Total
	✓	x	→	✓	x	→	✓	x	✓
Strength (10)	3	0	7	2 + 1↓	3	1	1	0	6
Endurance (14)	6	0	8	5 + 2↓	0	1	1	0	12
Coordination (9)	2	0	7	2 + 2↑	3	2	1 + 1↓	0	7
Balance (10)	5	0	5	3 + 2↑	2	0	2↑	0	12
Agility (15)	6	0	9	5 + 1↓	1	2	1 + 1↓	0	12
Flexibility (4)	2	0	2	2	0	0	0	0	4
Total (62)	24	0	38	19 + 4	9	6	6	0	53

*Note:* **✓** item accepted; **|x** item rejected; **| →** item moved to next round; **|↓** item moved to other domain; **|**↑ item added from another domain.

The major concern raised by the panellists in validating the items was the difficulty in following complex instructions, as most children with DS have mild to moderate intellectual disability. Panellists also suggested avoiding jumping and hopping activities, raising safety concerns about the risk of injury to activity performance owing to joint hypermobility and ligamental laxity. Some items were rejected because they were repetitive or similar to another activity in the same domain or another domain. Table 4 presents the list of items shifted or rejected during the validation process. Table 5 presents the I‐CVIs of the included items at the end of the three rounds of the consensus process.

**TABLE 4:** List of BOOST‐DS items rejected/shifted during the content validation process.

Domain	Items	Rejected (x) shifted (→)	Reason for (x)/(→)	Shifted domain
Strength	Leg wrestling	x	Risk of Injury	—
	Rabbit jumps	x	Jumping/Hopping	—
	Catch and throw	x	Complex Instructions	—
	Target	→	Focused Task	Coordination
Balance	Relay: hops and jumps	x	Jumping/Hopping	—
	Scavenger hunt	x	Chaos	—
Endurance	Reverse dodgeball	→	Focused Task	Coordination
	Single leg hops	→	Mobility	Balance
Coordination	Three‐legged race	x	Risk of Injury	—
	Hidden items	x	Complex Instructions	—
	Message received	x	Complex Instructions	—
	Gongee	→	Mobility	Balance
Agility	Kho‐Kho	x	Risk of Injury	—
	Queen of Shebaa	→	Mobility	Balance
	Ladders	→	Mobility	Balance

**TABLE 5:** Content validity of each item (I‐CVI).

Domains	Active play items	Consensus achieved in round	Relevance	Appropriateness	Clarity of instructions	Feasibility
Strength (6)	Monkey in the middle	2	0.9	0.9	0.9	0.9
	Kicks	1	0.9	0.8	0.9	0.8
	Frog jump	2	1.0	1.0	1.0	1.0
	Duck walk	3	0.9	0.8	0.9	0.9
	Military crawl	1	1.0	0.8	1.0	0.9
	Arm wrestling	1	0.9	0.8	1.0	0.9
Endurance (12)	Blind man's bluff	2	0.8	0.8	0.8	0.8
	Sea and shore	2	1.0	1.0	0.9	0.9
	Football	1	1.0	1.0	0.8	0.9
	Basketball	2	0.8	0.8	1.0	0.9
	Fire in the mountain	1	1.0	1.0	0.9	0.8
	Tag	1	1.0	1.0	1.0	1.0
	Cow and tiger	3	0.9	0.9	0.9	0.9
	Lock and key	1	0.9	0.8	0.8	0.8
	Chain cut	1	0.9	0.9	0.9	0.8
	Run in a circle	2	1.0	1.0	0.9	0.9
	Cut the cake	2	0.8	0.9	0.9	0.9
	Cricket	1	0.9	0.8	0.9	0.9
Coordination (7)	We move	2	0.9	0.8	0.8	0.8
	Head, shoulders, knee	2	1.0	1.0	1.0	1.0
	Passing the parcel	3	0.9	0.9	0.9	1
	Big fish small fish	1	1.0	0.9	0.8	0.8
	Dribble the ball	1	0.9	0.8	0.9	0.9
	Reverse dodgeball^a^	2	0.8	0.8	0.8	0.9
	Target^a^	2	0.8	0.8	0.9	0.8
Balance (12)	Side shuffles	1	0.9	0.9	0.9	0.8
	Crocodile can I cross the river	2	1.0	1.0	1.0	1.0
	in the pond, on the bank	2	1.0	1.0	1.0	0.9
	Relay‐book balancing	1	0.8	0.9	1.0	0.8
	Relay‐lemon and spoon	1	1.0	0.9	1.0	0.9
	Relay‐balloon behind the back	1	0.9	0.8	0.9	0.9
	Walking in lines	1	1.0	1.0	1.0	1.0
	Hopscotch	2	0.8	0.8	0.9	0.8
	Single leg hops^a^	2	0.8	0.8	0.9	0.9
	Queen of sheeba^a^	2	0.8	0.8	0.8	0.9
	Ladders^a^	3	0.8	0.8	0.8	0.9
	Gongee^a^	3	1	1	1	1
Agility (12)	Dodgeball	1	0.8	0.8	0.9	0.9
	Dog and the bone	1	0.9	0.9	0.9	0.9
	Zig‐zag	1	1.0	1.0	1.0	1.0
	t‐drills	1	0.9	0.9	0.9	0.8
	Red light, green light	3	0.9	1.0	1.0	0.8
	Prisoners	2	0.8	0.8	0.9	0.8
	Colour colour	2	0.9	0.9	0.9	0.9
	Simon says	1	1.0	1.0	1.0	1.0
	Balloon up	2	0.8	0.8	0.8	0.8
	Musical chairs	1	0.9	0.9	0.9	0.9
	Musical chairs with numbers	2	0.8	0.8	0.8	0.9
	Crows and cranes	2	0.8	0.9	0.8	0.8
Flexibility (4)	Hula hoops	2	0.8	0.9	0.9	0.8
	Limbo	1	0.9	0.9	1.0	1.0
	Copy me	1	1.0	0.9	1.0	0.9
	Mountain and valleys	2	0.8	0.9	0.9	0.8

^a^ Item added from another domain.

## 4 Discussion

Intellectual impairment is one of the nonmodifiable negative correlates of physical activity (Li et al. 2016), and it can affect children with DS's potential to participate in physical activity. Nevertheless, participation can be improved by enhancing psychological factors (such as preferences, motivation and performance satisfaction), which are modifiable positive correlates of physical activity in children with and without disabilities (Li et al. 2016; Sallis et al. 2000). Therefore, during the design phase of the study, the investigators adopted and extended the participation construct framework (Imms et al. 2016) and integrated it with the SAAFE teaching principles (Lubans et al. 2017) to design a conceptual model for the optimal physical activity participation of children with DS with *intellectual disabilities*. Our conceptual model is similar to a multidimensional model (Kang et al. 2014), which has identified the key dimensions and determinants of optimal physical activity participation for children with *physical disabilities*. The model proposes that interactions between dimensions and determinants and their dynamic balance can impact the health, well‐being and QoL of children with physical disabilities (Kang et al. 2014). A systematic review by Ross et al. (2016) examined conceptual and methodological approaches for evaluating physical activity participation in children with disabilities and distinguished between physical activity engagement and participation. Ross et al. (2016) offer a transcending approach in conceptualising physical activity engagement beyond the context (involving attendance and difficulty levels of activity) to physical activity participation, which includes performance (sense of achievement) and participation outcomes (fun, enjoyment, a sense of inclusivity and self‐efficacy). Furthermore, they advocate that physical activity should be considered a context in which participation can be enhanced through dynamic interactions between the child and the environment, resulting in a quality health experience. Our conceptual model aligns with the proposal by Ross et al. (2016), which emphasises the design of physical activities to enhance participation rather than merely engagement.

During the study's development phase, the investigators focused on implementing active play that does not require sophisticated equipment or resources in a special school setting for children with DS. The majority of special schools for children with disabilities in India are run by nongovernmental organisations with very limited resources (Kumar et al. 2012). Thus, implementing rehabilitation and health services for children with special needs remains a challenge (World Health Organisation and World Bank 2011). Given this challenge, the investigators considered implementing a play‐based physical activity programme to improve overall health outcomes for children with DS. Through a systematic literature search, the investigators identified six domains of physical activity programme—strength, endurance, flexibility, coordination, balance and agility (Jacinto et al. 2021; Kashi et al. 2023; Lin and Wuang 2012; Rodríguez‐Grande et al. 2022; Sugimoto et al. 2016). However, only a limited number of studies have reported physical activity programmes based on play or games (Johnstone et al. 2019; McGarty et al. 2021; Priyono et al. 2021). Therefore, the investigators used a rapid ideation brainstorming technique to identify active play items that could potentially enhance physical activity participation among children with DS. The effectiveness of such processes of ideation (divergent thinking) and iteration (convergent thinking) has been embedded in the ‘design thinking’ approach (Wolcott et al. 2021), which is increasingly being adapted in healthcare research (Altman et al. 2018; Ferreira et al. 2015; Georgiadis 2021; Marra et al. 2018; Milroy et al. 2021). As a result, of the 83 active play items identified through the individual ideation process, 62 items were narrowed down through the group iteration process. Thus, the BOOST‐DS programme, comprising 62 active play items, was identified across six domains of physical activity.

The investigators used the modified Delphi consensus approach to validate the contents of the BOOST‐DS programme. Among the 62 items, the expert panel reached a consensus on the contents of 53 items across the domains of strength (6 items), endurance (12 items), balance (10 items), coordination (7 items), agility (14 items) and flexibility (4 items). The content‐validated BOOST‐DS programme prototype aims to improve fundamental motor skills and enhance physical activity participation in children with DS.

The present study has strengths and limitations. The BOOST‐DS programme is dynamic, flexible and comprehensive, encompassing all domains of physical activity to enhance the fundamental motor skills of children with DS. Through creativity and innovation, more active play items can be incorporated into the BOOST‐DS programme. Implementing the BOOST‐DS programme in the community‐based special school setting could potentially enhance active play participation, thereby increasing the opportunities to improve the fundamental motor and cognitive skills of children with DS. Furthermore, most active play items involve more than one child. The facilitators can group children with DS into teams and organise active play as competitive games. Thus, collaborative participation and peer group interactions can enhance the social negotiation skills of the children with DS. Our programme can be considered a cost‐effective method for improving overall health outcomes (Sarkar and Mateus 2022) in children with DS through participation‐focused physical activity programmes in resource‐constrained settings such as schools. Our BOOST‐DS programme shares the characteristics of frugal innovation in several ways (Khan 2016; Weyrauch and Herstatt 2016). The BOOST‐DS programme has been modelled on established theories and principles (robustness) of participation‐focused physical activity in children. Therapists can implement the programme to motivate physical activity participation in children with DS (user‐friendliness), involving minimal resources (affordability), in a school environment (accessibility). The BOOST‐DS programme can be used by therapists to train special educators to adapt physical activity participation in routine games and leisure activities (sustainability and scalability), which could potentially enhance the participation outcomes of children with DS (attractive value proposition) (Khan 2016; Weyrauch and Herstatt 2016).

The study is a self‐funded postgraduate dissertation conducted within a specific time frame. For this reason, the investigators could embed only a few elements of design thinking (Altman et al. 2018) in the process of designing, developing, and validating the contents of the BOOST‐DS programme. While the investigators systematically generated active play items through ideation, prototyped them through iterations and content‐validated them through expert consensus, the investigators did not formally conduct interviews with relevant stakeholders to identify the need for the study. The investigators used an informal approach to determine the study need, drawing on anecdotal experiences of teachers and parents and directly observing children with DS during the postgraduate students' training opportunities in special school settings. Furthermore, the investigators could not test the feasibility of the BOOST‐DS programme to evaluate children with DS as end‐users. While design thinking is considered a strategic tool for implementing physical activity programmes for children with disabilities (Marra et al. 2018), the investigators did not involve physical educators, parents, teachers or children with DS in co‐designing the BOOST‐DS programme due to logistic constraints.

## 5 Conclusion

The investigators designed, developed and content‐validated the 53‐item BOOST‐DS programme, which focuses on active play‐based physical activities across six domains of motor skills. The BOOST‐DS programme can potentially enhance participation outcomes, encompassing improvements in fundamental motor skills, cognitive skills, and social negotiation skills in children with DS. The investigators suggest implementing the BOOST‐DS programme, which aligns with the World Health Organisation 2020 guidelines on physical activity recommendations for children (Bull et al. 2020). The WHO 2020 guidelines strongly recommend implementing three to five sessions of moderate‐to‐vigorous aerobic physical activity for children, combining aerobic and strength training for at least 60 min/day across the week. While this recommended dosage may not be achievable for children with disabilities, the overall recommendation is to reduce time spent in sedentary behaviour (Bull et al. 2020). Therefore, for children with DS, two to three active play items on each of the strength and endurance domains of the BOOST‐DS programme can be encouraged for at least 30 min/day and two to three items each on balance, coordination, agility and flexibility domains on rotation can be encouraged for an additional 15 min/day for three to five sessions across the week depending on their individual abilities. The active play items across six domains of the BOOST‐DS offer many choices and variety children with DS to participate in physical activities, which could facilitate achieving the recommended physical activity levels.

While the present study established the content validity of the BOOST‐DS programme, future research should evaluate its feasibility and effectiveness through pilot implementations. Suggested approaches include (1) a mixed‐methods feasibility study with stakeholders (special educators, physical educators, parents, children with DS and school administration) to refine delivery protocols, (2) an A‐B‐A‐B design that compares BOOST‐DS to standard physical education programmes to assess improvements in the levels of participation, fundamental motor skills, cognitive skills and social negotiation skills and (3) longitudinal studies tracking health impacts (overall health outcomes, physical fitness and participation) of the BOOST‐DS programme. These steps strengthen the evidence for real‐world adoption of the BOOST‐DS programme.

## Funding

The authors have nothing to report.

## Ethics Statement

This study was approved by the Institution Research Committee, Manipal College of Health Professions (approval no. MCHP‐Mpl/IRC/PG/2023/063) on 25 February 2023. This study received ethical approval from the Kasturba Medical College and Kasturba Hospital Institution Ethics Committee (approval no. IEC2: 234/2023) on 14 April 2023.

## Conflicts of Interest

The authors declare no conflicts of interest.

## Supporting information




**Data S1:** Supporting information.

## Data Availability

The materials supporting the study administration and the data supporting the results and analyses of the consensus process are provided as a Supporting Information.
