# Supplementary material for: Design and Development of ‘Building Opportunities for Optimal physical activity Skills Training in children with Down Syndrome (BOOST‐DS)’ Programme and its Content Validation through Consensus using Modified Delphi method
Source: J Intellect Disabil Res. 2026 May 22;70(8):811–22. doi: 10.1111/jir.70119 (PMC13327175; doi:10.1111/jir.70119)
Supplement: Supplementary file 1 — Data S1: Supporting information. [file JIR-70-811-s001.docx]

**Design and Development of 'Building Opportunities for Optimal physical activity Skills Training in children with Down Syndrome (BOOST-DS)’ Program and its Content Validation through consensus using modified Delphi method**

**SUPPLEMENTARY FILES**

**S1.** ACCORD (Accurate Consensus Reporting Document) Checklist

| Item No. | Section | Checklist Item (*help text*) | Page No. |
| --- | --- | --- | --- |
| T1 | **Title** | Identify the article as reporting a consensus exercise and state the consensus methods used in the title.  *For example, Delphi or nominal group technique.* | 1 |
| I1 | **Introduction** | Explain why a consensus exercise was chosen over other approaches. | 5 |
| I2 |  | State the aim of the consensus exercise, including its intended audience and geographical scope (national, regional, global). | 5 |
| I3 |  | If the consensus exercise is an update of an existing document, state why an update is needed, and provide the citation for the original document. | NA |
| M1 | **Methods**  Registration | If the study or study protocol was prospectively registered, state the registration platform and provide a link. If the exercise was not registered, this should be stated.  *Recommended to include the date of registration.* | 8 |
| M2 | Selection of SC and/or panellists | Describe the role(s) and areas of expertise or experience of those directing the consensus exercise.  *For example, whether the project was led by a chair, co-chairs or a steering committee, and, if so, how they were chosen. List their names if appropriate, and whether there were any subgroups for individual steps in the process.* | 10 |
| M3 |  | Explain the criteria for panellist inclusion and the rationale for panellist numbers. State who was responsible for panellist selection. | 10 |
| M4 |  | Describe the recruitment process (how panellists were invited to participate).  *Include communication/advertisement method(s) and locations, numbers of invitations sent, and whether there was centralised oversight of invitations or if panellists were asked/allowed to suggest other members of the panel.* | 10 |
| M5 |  | Describe the role of any members of the public, patients or carers in the different steps of the study. | NA |
| M6 | Preparatory research | Describe how information was obtained prior to generating items or other materials used during the consensus exercise.  *This might include a literature review, interviews, surveys, or another process.* | 8-9 |
| M7 |  | Describe any systematic literature search in detail, including the search strategy and dates of search or the citation if published already.  *Provide the details suggested by the reporting guideline PRISMA and the related PRISMA-Search extension.* | 8  S2 |
| M8 |  | Describe how any existing scientific evidence was summarised and if this evidence was provided to the panellists. | 6-8  conceptual model not shared with panellists |
| M9 | Assessing consensus | Describe the methods used and steps taken to gather panellist input and reach consensus (for example, Delphi, RAND-UCLA, nominal group technique).  *If modifications were made to the method in its original form, provide a detailed explanation of how the method was adjusted and why this was necessary for the purpose of your consensus-based study.* | 10-11 |
| M10 |  | Describe how each question or statement was presented and the response options. State whether panellists were able to or required to explain their responses, and whether they could propose new items.  *Where possible, present the questionnaire or list of statements as supplementary material.* | 10-11  S3 |
| M11 |  | State the objective of each consensus step.  *A step could be a consensus meeting, a discussion or interview session, or a Delphi round.* | 10 |
| M12 |  | State the definition of consensus (for example, number, percentage, or categorical rating, such as ‘agree’ or ‘strongly agree’) and explain the rationale for that definition. | 11 |
| M13 |  | State whether items that met the prespecified definition of consensus were included in any subsequent voting rounds. | 11 |
| M14 |  | For each step, describe how responses were collected, and whether responses were collected in a group setting or individually. | 11 |
| M15 |  | Describe how responses were processed and/or synthesised.  *Include qualitative analyses of free-text responses (for example, thematic, content or cluster analysis) and/or quantitative analytical methods, if used.* | 11 |
| M16 |  | Describe any piloting of the study materials and/or survey instruments.  *Include how many individuals piloted the study materials, the rationale for the selection of those individuals, any changes made as a result and whether their responses were used in the calculation of the final consensus. If no pilot was conducted, this should be stated.* | Piloting not done |
| M17 |  | If applicable, describe how feedback was provided to panellists at the end of each consensus step or meeting.  *State whether feedback was quantitative (for example, approval rates per topic/item) and/or qualitative (for example, comments, or lists of approved items), and whether it was anonymised.* | 11  S5 |
| M18 |  | State whether anonymity was planned in the study design. Explain where and to whom it was applied and what methods were used to guarantee anonymity. | 10 |
| M19 |  | State if the steering committee was involved in the decisions made by the consensus panel.  *For example, whether the steering committee or those managing consensus also had voting rights.* | NA |
| M20 | Participation | Describe any incentives used to encourage responses or participation in the consensus process.  *For example, were invitations to participate reiterated, or were participants reimbursed for their time.* | NA |
| M21 |  | Describe any adaptations to make the surveys/meetings more accessible.  *For example, the languages in which the surveys/meetings were conducted and whether translations or plain language summaries were available*. | NA |
| R1 | Results | State when the consensus exercise was conducted. List the date of initiation and the time taken to complete each consensus step, analysis, and any extensions or delays in the analysis. | 11 |
| R2 |  | Explain any deviations from the study protocol, and why these were necessary.  *For example, addition of panel members during the exercise, number of consensus steps, stopping criteria; report the step(s) in which this occurred.* | NA  No deviations |
| R3 |  | For each step, report quantitative (number of panellists, response rate) and qualitative (relevant socio-demographics) data to describe the participating panellists. | 12-13 / 32 |
| R4 |  | Report the final outcome of the consensus process as qualitative (for example, aggregated themes from comments) and/or quantitative (for example, summary statistics, score means, medians and/or ranges) data. | 12-13 / 33-36 |
| R5 |  | List any items or topics that were modified or removed during the consensus process. Include why and when in the process they were modified or removed. | 33-34 |
| D1 | Discussion | Discuss the methodological strengths and limitations of the consensus exercise.  *Include factors that may have impacted the decisions (for example, response rates, representativeness of the panel, potential for feedback during consensus to bias responses, potential impact of any non-anonymised interactions).* | 15-17 |
| D2 |  | Discuss whether the recommendations are consistent with any pre-existing literature and, if not, propose reasons why this process may have arrived at alternative conclusions. | NA |
| O1 | Other information | List any endorsing organisations involved and their role. | NA |
| O2 |  | State any potential conflicts of interests, including among those directing the consensus study and panellists. Describe how conflicts of interest were managed. | 17 |
| O3 |  | State any funding received and the role of the funder.  *Specify, for example, any funder involvement in the study concept/design, participation in the steering committee, conducting the consensus process, funding of any medical writing support. This could be disclosed in the methods or in the relevant transparency section of the manuscript. Where a funder did not play a role in the process or influence the decisions reached, this should be specified.* | 17 |

From: PLoS Med 21(1): e1004326. <https://doi.org/10.1371/journal.pmed.1004326> For more information see: <https://www.ismpp.org/accord>

**S2.** Search Strategy

(("Down syndrome" OR "Trisomy 21”))

AND

((“Children”) OR (“child”) OR (“adolescents”) OR (“young adults”) OR (“youth”))

AND

((“exercise program") OR (“exercise programme”) OR (“physical activity program”) OR ("school-based physical therapy”) OR (“group therapy”) OR (“school-based physical activity interventions”) OR ("special school") OR ("after school”) OR ("before school") OR (“play”) OR (“play-based intervention”) OR (“play-based therapy”) OR ("training"))

Inclusion criteria: Articles that focus on children and adolescents with Down syndrome; Age: 5 to 18 years; Intervention: play based physical activity program/ physical activity program/ group exercise program/ individual exercise program with minimal equipment use.

Exclusion criteria: Interventions that use expensive equipment (treadmill, VR, Aquatic therapy, isokinetic training device) or specialised training/ equipment

Duration: Until December 31, 2023 | Filters: None

PubMed: 762

After title screening: 25

After full text screening: 8 included

**S3.** List of 83 play items mapped to physical activity (PA) domains, which were reviewed for suitability using four criteria

| **Item No.** | **Activity Name** | **PA Domain** | **Task complexity** | **Cognitive demand** | **Need for additional equipment** | **Safety concerns** | **Included** |
| --- | --- | --- | --- | --- | --- | --- | --- |
| 1 | monkey in the middle | strength | No | No | No | No | ✓ |
| 2 | volleyball | strength | No | No | **Yes** | **Yes** | x |
| 3 | kicking the ball | strength | No | No | No | No | ✓ |
| 4 | frog jump | strength | No | No | No | No | ✓ |
| 5 | rabbit jump | strength | No | No | No | No | ✓ |
| 6 | military crawl | strength | No | No | No | No | ✓ |
| 7 | catch and throw | strength | No | No | No | No | ✓ |
| 8 | relay- paper fish | strength | **Yes** | No | No | No | x |
| 9 | relay- wheelbarrow | strength | No | No | No | **Yes** | x |
| 10 | relay- picking paper cup with a balloon | strength | **Yes** | No | **Yes** | No | x |
| 11 | relay- target throwing | strength | No | No | No | No | ✓ |
| 12 | arm wrestling | strength | No | No | No | No | ✓ |
| 13 | leg wrestling | strength | No | No | No | No | ✓ |
| 14 | Duck walk | strength | No | No | No | No | ✓ |
| 15 | ddakji | strength | **Yes** | **Yes** | No | No | x |
| 16 | Blind mans bluff | endurance | No | No | No | No | ✓ |
| 17 | Fire in the mountain | endurance | No | No | No | No | ✓ |
| 18 | Reverse dodgeball | endurance | No | No | No | No | ✓ |
| 19 | Sea shore | endurance | No | No | No | No | ✓ |
| 20 | Football | endurance | No | No | No | No | ✓ |
| 21 | Basketball | endurance | No | No | No | No | ✓ |
| 22 | Tag | endurance | No | No | No | No | ✓ |
| 23 | cow and the tiger | endurance | No | No | No | No | ✓ |
| 24 | Single leg hops | endurance | No | No | No | No | ✓ |
| 25 | Lock and key | endurance | No | No | No | No | ✓ |
| 26 | Chain cut | endurance | No | No | No | No | ✓ |
| 27 | Run in a circle | endurance | No | No | No | No | ✓ |
| 28 | Cut the cake | endurance | No | No | No | No | ✓ |
| 29 | Kabaddi | endurance | **Yes** | **Yes** | No | **Yes** | x |
| 30 | Cricket | endurance | No | No | No | No | ✓ |
| 31 | Footloose/crazy legs | flexibility | No | No | No | **Yes** | x |
| 32 | Relay- mountains and valleys | flexibility | No | No | No | No | ✓ |
| 33 | Relay- dressing | flexibility | **Yes** | **Yes** | **Yes** | No | x |
| 34 | Passing a hula hoop | flexibility | No | No | No | No | ✓ |
| 35 | Copy me! | flexibility | No | No | No | No | ✓ |
| 36 | Doctor Doctor | flexibility | **Yes** | **Yes** | **Yes** | **Yes** | x |
| 37 | Limbo | flexibility | No | No | No | No | ✓ |
| 38 | action songs- grand old duke of york | coordination | **Yes** | **Yes** | **Yes** | No | x |
| 39 | action songs- o chester | coordination | **Yes** | **Yes** | **Yes** | No | x |
| 40 | action songs- we move to the left | coordination | No | No | No | No | ✓ |
| 41 | action songs- head shoulders knees and toes | coordination | No | No | No | No | ✓ |
| 42 | action songs- hokey pokey | coordination | No | **Yes** | No | No | x |
| 43 | passing the parcel | coordination | No | No | No | No | ✓ |
| 44 | big fish small fish | coordination | No | No | No | No | ✓ |
| 45 | relay- three legged race | coordination | No | No | No | No | ✓ |
| 46 | relay- find the hidden items | coordination | No | No | No | No | ✓ |
| 47 | goti | coordination | No | No | **Yes** | **Yes** | x |
| 48 | gongee | coordination | No | No | No | No | ✓ |
| 49 | message received | coordination | No | No | No | No | ✓ |
| 50 | dribbling a ball | coordination | No | No | No | No | ✓ |
| 51 | memory game | coordination | No | **Yes** | No | No | x |
| 52 | origami | coordination | **Yes** | **Yes** | **Yes** | No | x |
| 53 | Side shuffle while catching and throwing a ball | balance | No | No | No | No | ✓ |
| 54 | Crocodile can I cross the river | balance | No | No | No | No | ✓ |
| 55 | In the pond, on the bank | balance | No | No | No | No | ✓ |
| 56 | Relay- stepping stones | balance | No | No | No | **Yes** | x |
| 57 | Relay- book balancing | balance | No | No | No | No | ✓ |
| 58 | Relay- lemon and spoon | balance | No | No | No | No | ✓ |
| 59 | Relay- balloon behind the back | balance | No | No | No | No | ✓ |
| 60 | Hopskotch | balance | No | No | No | No | ✓ |
| 61 | Scavenger hunt | balance | No | No | No | No | ✓ |
| 62 | Walking in lines | balance | No | No | No | No | ✓ |
| 63 | dodgeball | agility | No | No | No | No | ✓ |
| 64 | dog and the bone | agility | No | No | No | No | ✓ |
| 65 | head,shoulders, knees | agility | No | No | No | No | ✓ |
| 66 | zigzag running | agility | No | No | No | No | ✓ |
| 67 | t drills | agility | No | No | No | No | ✓ |
| 68 | red light green light | agility | No | No | No | No | ✓ |
| 69 | prisoners | agility | No | No | No | No | ✓ |
| 70 | action songs- in and out the sparkling waters | agility | **Yes** | **Yes** | No | No | x |
| 71 | action songs- oranges and lemons | agility | **Yes** | **Yes** | No | No | x |
| 72 | colour colour | agility | No | No | No | No | ✓ |
| 73 | queen of sheba | agility | No | No | No | No | ✓ |
| 74 | simon says | agility | No | No | No | No | ✓ |
| 75 | keep the balloon up | agility | No | No | No | No | ✓ |
| 76 | goldsport | agility | **Yes** | **Yes** | No | **Yes** | x |
| 77 | ladders | agility | No | No | No | No | ✓ |
| 78 | musical chairs | agility | No | No | No | No | ✓ |
| 79 | floor is lava | agility | No | No | No | **Yes** | x |
| 80 | musical chairs with numbers | agility | No | No | No | No | ✓ |
| 81 | musical chairs with an activity on each chair | agility | **Yes** | **Yes** | No | No | x |
| 82 | crows and cranes | agility | No | No | No | No | ✓ |
| 83 | kho kho | agility | No | No | No | No | ✓ |

**S4.** Validation Questionnaire used for Expert Rating on a Likert Scale

Likert Scale: 1. Strongly Disagree; 2. Disagree; 3. Unsure; 4. Agree; 5. Strongly Agree

| **ITEM NO.** | **ITEMS** | **ACTIVITY** | **ACTIVITY DESCRIPTION** | **RELEVANCE** | **APPROPRIATENESS** | **CLARITY OF INSTRUCTIONS** | **FEASIBILITY** | **Reviewer Decision** | | |
| --- | --- | --- | --- | --- | --- | --- | --- | --- | --- | --- |
|  |  |  |  | **Is the activity focusing on the “component of physical activity” it is intended to focus on?** | **Is the activity applicable / performable by children with Down syndrome?** | **Are the instructions clear and simple for children with Down syndrome to follow?** | **Is the activity easy to perform in a special school setting for children with Down syndrome?** | **Accept** | **Accept with modifications** | **Reject** |
|  |  |  |  | **1-5** | **1-5** | **1-5** | **1-5** |  |  |  |
| **PA DOMAIN:** **STRENGTH** | | |  |  |  |  |  |  |  |  |
| 1 | Monkey in the middle | S1 | One person stands in the middle of a circle (monkey). The other children form a circle and they have to throw the ball to each other and not allow the person in the middle to catch the ball. If the monkey catches the ball then the person who threw the ball becomes the next monkey. |  |  |  |  |  |  |  |
| 2 | Kicks | S2 | Kick the ball into the goal post, either facing the goal post or facing to the side and kicking sideways into the goal post. Each child gets 5 or 10 chances. Total goals are counted. |  |  |  |  |  |  |  |
| 3 | Frog Jump | S3 | Children have to be in full squat with hands on the ground, they have to push on the ground with all four limbs and jump to go ahead. They have to to do this from starting to ending point (like a race) |  |  |  |  |  |  |  |
| 4 | Rabbit Jump | S4 | Children have to half squat and jump. They must do this from starting to ending point (like a race) |  |  |  |  |  |  |  |
| 5 | Military crawl | S5 | Children have to crawl on forearms while dragging their lower body forwards. They must do this from starting to ending point (like a race) |  |  |  |  |  |  |  |
| 6 | Catch & throw | S6 | Children stand in a circle. The children catch and throw a ball to each other/ to the therapist. (Therapist can stand in the centre of the circle) |  |  |  |  |  |  |  |
| 7 | Target | S7 | There are two teams who are standing opposite to each other at a distance. There is mark on the floor halfway between the starting point of both the teams, where a bucket is placed. The person standing in the first aims and tries to throw the ball into the bucket as many times as possible for 10 seconds and runs back and joins the line. The next child in line continues the same. |  |  |  |  |  |  |  |
| 8 | Arm Wrestling | S8 | Two individuals sit across the table and hold the other person's hand with their elbows on the table. They try to push each other's hand down, to the side to touch the table. |  |  |  |  |  |  |  |
| 9 | Leg Wrestling | S9 | Two people lie on the mat and intwine one of their legs. On the count of three, each one has to try to push the other person's leg down and make it touch the ground. Whoever makes the other person's leg touch the floor, wins the game. |  |  |  |  |  |  |  |
| 10 | Duck Walk | S10 | Children do a complete squat and then have to walk while squatting. They must do this from starting to ending point (like a race) |  |  |  |  |  |  |  |
| **PA DOMAIN:** **ENDURANCE** | | |  |  |  |  |  |  |  |  |
| 11 | Blind man's bluff | E1 | One person (teacher/ therapist) blind folds themselves and attempts to catch the children while they try to run around and avoid getting caught by the blindfolded person. Another person (teacher/therapist) can take care of the safety of the children and the blindfolded person |  |  |  |  |  |  |  |
| 12 | fire in the mountain | E2 | Children run in a circle (holding hands) singing "fire in the mountain, run, run, run" while the person in the middle shouts a random number, the children have to break the circle and form groups of that specific number that was shouted. Whoever could not form the specific group are considered 'out'. The game continues by forming the circle again. Game is played until last one/two children are left. |  |  |  |  |  |  |  |
| 13 | reverse dodgeball | E3 | One person (therapist/teacher) stand in the middle of the circle, the other children form a circle around and stand at a distance. the person in the middle who has to throw the ball below the knee level on the children forming the circle and get them out of the game. The children forming the circle have to dodge the ball either by jumping or moving away |  |  |  |  |  |  |  |
| 14 | sea and shore | E4 | One side of the room/ ground is designated as sea, the opposite side as shore. The person conducting the games gives command: 'sea' (run to the side designated as sea); 'shore' (run to the side designated as shore); 'rains falling' (squat and sit with hands on the head); 'captains calling' (stand and salute); 'freeze' (freeze in whatever position you currently are).  Whoever makes a mistake in following the commands is out of the game and has to sit on the sides. Continue until last few children (4-5) are left |  |  |  |  |  |  |  |
| 15 | football | E5 | Children will be divided into two groups. The aim of the game is to aim and kick the ball into the goalpost (giving one goal/ point to that team). The teams will compete to run and get the ball and kick it onto the goalpost to make a goal |  |  |  |  |  |  |  |
| 16 | basketball | E6 | Children will be divided into two groups, the children stand in a line and one after the other. They have to dribble the ball 3 times and then have to aim the ball and deliver it into the basket (typical basketball basket). Each child will get 3 chances. They compete for whichever team puts more baskets. |  |  |  |  |  |  |  |
| 17 | tag | E7 | There will be a denner (one of the children) who will run and catch the other children. The other children will run in the given space without getting caught. If they are touched by the denner then they are considered out and they become the next denner. |  |  |  |  |  |  |  |
| 18 | cow and tiger | E8 | All children will be made to stand in a circle while holding hands, two children will be selected of which one will be a tiger and one will be a cow. The tiger will to be outside the circle and cow will be inside the circle. The cow has to run away from the tiger and can freely go under the hands of the children forming the circle. But the tiger needs permission (from the children forming the circle) to go inside or outside. When he gets the permission, the tiger runs and tries to catch the cow.  If the cow gets caught then the therapist decides who will be the tiger and the cow for the next round. If the cow does not get caught then the therapists changes the cow and the tiger after 1-2mins of running |  |  |  |  |  |  |  |
| 19 | single leg hops | E9 | One child will be the denner. The denner has to hop on single leg and catch the other players who are running in the given space without getting caught. The child who gets caught is the next denner. |  |  |  |  |  |  |  |
| **ITEM NO.** | **ITEMS** | **ACTIVITY** | **ACTIVITY DESCRIPTION** | **RELEVANCE** | **APPROPRIATENESS** | **CLARITY OF INSTRUCTIONS** | **FEASIBILITY** | **Reviewer Decision** | | |
|  |  |  |  | **Is the activity focusing on the “component of physical activity” it is intended to focus on?** | **Is the activity applicable / performable by children with Down syndrome?** | **Are the instructions clear and simple for children with Down syndrome to follow?** | **Is the activity easy to perform in a special school setting for children with Down syndrome?** | **Accept** | **Accept with modifications** | **Reject** |
|  |  |  |  | **1-5** | **1-5** | **1-5** | **1-5** |  |  |  |
| 20 | lock and key | E10 | There will be one denner. All other children are running in a given space. If the denner touches any player, they will get 'lock'ed and that player has to stand in the same spot and cannot run.  The other players can release the ones who are 'lock'ed by touching them and giving them a 'key', then they can start running again. The children have to try to run around and escape from getting 'lock'ed. If a child who is 'lock'ed takes a step/runs without getting a 'key'; he becomes a denner. |  |  |  |  |  |  |  |
| 21 | chain cut | E11 | There will be a single denner at the beginning of the game, who will start by catching one player. Then, both of them will hold hands and catch another player at random. Each time one person is caught, they will hold hands of the previously caught players and catch the next person. The chain continues to grow till the last person is caught. The chain cannot break while running, if it does then the two people who broke the chain start from the beginning and catch people to increase the chain. |  |  |  |  |  |  |  |
| 22 | run in a circle | E12 | All children will stand in a circle. The therapist will give the following instructions- 'If I blow the whistle once you have to run clockwise, if I blow the whistle twice you have to run anticlockwise and if I blow the whistle thrice you stand still'.  Whoever makes a mistake in following the command, gets out of the game and sits. |  |  |  |  |  |  |  |
| 23 | cut the cake | E13 | Everyone stands in a circle holding hands. One pair runs outside the circle and touches the pair of hands they want to challenge. The two people whose interlocked hands have been touched have to run. |  |  |  |  |  |  |  |
| 24 | cricket | E14 | Divide them into 2 groups and play the traditional game of cricket. (or) Give each child a chance to bat for 5 balls and then the other children can take chances at throwing the ball or doing fielding. |  |  |  |  |  |  |  |
| **PA DOMAIN:** **CO-ORDINATION** | | |  |  |  |  |  |  |  |  |
| 25 | We move | C1 | Everyone stands in a circle. The song is sung and they have to follow the following commands and move accordingly: 'we move to the left' (side shuffle two spaces to the left)  'we move to the right' (side shuffle two spaces to the right) 'we move and move' (side shuffle two places to the left)  'move all night' (side shuffle two places to the right) 'heel and toe' (point heel and toe alternately)  'half way go' (turn halfway)  'a new friend is here' (spin/ turn around and point to the person standing next to them)" |  |  |  |  |  |  |  |
| 26 | head, shoulders, knee | C2 | Two people stand on either sides of a cone. They have to touch their head, shoulders or knees as per the commands given by the person conducting the game (therapist/teacher). When the person conducting gives the command 'catch' they have to quickly grab the cone. The first to grab the cone gets the point |  |  |  |  |  |  |  |
| 27 | passing the parcel | C3 | Everyone stands in a circle, music is played. The therapist gives them an object to be passed around while playing music. Once the music stops the person with the object in hand has to perform a task (Example:10 squats, 5 lunges, dance for 30 seconds, 7 jumps, etc.,). |  |  |  |  |  |  |  |
| 28 | big fish small fish | C4 | The therapist will give commands of "big fish" where the children have to hold their arms out at a wide distance to show the size of the fish, and "small fish" where they will reduce the distance between their hands to depict a small fish. The commands get faster with time. |  |  |  |  |  |  |  |
| 29 | three legged race | C5 | It is a race to be done in pairs. One person will tie their left leg to the right leg of the other person in the team and they both have to coordinate their leg movements and run to the finish line. |  |  |  |  |  |  |  |
| 30 | hidden items | C6 | One person will run from each team to the therapist who will show them a picture which can be any sort of puzzle such as find the difference/find the hidden item/find the hidden word. Each person finds and circles one item and runs back to their team. First team to find all the items wins. |  |  |  |  |  |  |  |
| 31 | gongee | C7 | Children will be given five stones, he has to first throw the stones on the ground. He has to stand with one leg behind the other (Tandem standing) and pick up the stones. If the stones are apart, he can walk with one leg behind the other (tandem walking) and pick them. |  |  |  |  |  |  |  |
| 32 | message receieved | C8 | Everyone sits in a circle holding hands (hook grasp) with the denner (Child or therapist) in the middle. The game starts from one person in the circle who says I want to pass my message to 'x' (name of any child).  After saying that, the person gently presses the hand of the person sitting on any one side (he/she can press the hands of one person only, either left or right side). That person, then passes the message forward by gentling pressing the hand of the person next to him, This continues such that the message reaches 'x'. Once 'x' receives the message he has to say 'message received'.  The denner has to observe the hand movements while the message travels from the sender to the recipient and if he observes any hand movement then he has to call out their name and they become the next denner. |  |  |  |  |  |  |  |
| 33 | dribble the ball | C9 | Child has to dribble the ball on the floor and run from one end of the room to another |  |  |  |  |  |  |  |
| BALANCE | | |  |  |  |  |  |  |  |  |
| 34 | side shuffles | B1 | Two people stand opposite each other throwing and catching a ball while walking side ways |  |  |  |  |  |  |  |
| 35 | crocodile can I cross the river | B2 | The denner is the therapist. The players have to ask the denner if they can cross the river, if he says yes then they jump forward, if he says no then they have to jump back once |  |  |  |  |  |  |  |
| 36 | in the pond, on the bank | B3 | A line will be drawn on the floor. The therapist will give commands of "in the pond" then the children jump forward or "on the bank" the stay on this side of the river/ jump back (in case they are on the other side of the pond) |  |  |  |  |  |  |  |
| **ITEM NO.** | **ITEMS** | **ACTIVITY** | **ACTIVITY DESCRIPTION** | **RELEVANCE** | **APPROPRIATENESS** | **CLARITY OF INSTRUCTIONS** | **FEASIBILITY** | **Reviewer Decision** | | |
|  |  |  |  | **Is the activity focusing on the “component of physical activity” it is intended to focus on?** | **Is the activity applicable / performable by children with Down syndrome?** | **Are the instructions clear and simple for children with Down syndrome to follow?** | **Is the activity easy to perform in a special school setting for children with Down syndrome?** | **Accept** | **Accept with modifications** | **Reject** |
|  |  |  |  | **1-5** | **1-5** | **1-5** | **1-5** |  |  |  |
| 37 | relay-book balancing | B4 | Children are divided into teams. (3 to 5 teams depending on the total number of children). It is a race, where the child has to balance a book on the head (should not touch/hold the book with hands) and reach the therapist and then hold the book in his hand and run back to the team. Then the next member in the team can repeat the same. Team that finishes the relay first, wins. |  |  |  |  |  |  |  |
| 38 | relay-lemon and spoon | B5 | Place the behind part of a spoon in your mouth, place a lemon on the spoon and race to a finish line. It can be done in slots (7-8 children per slot) and winners can be identified for each slot. |  |  |  |  |  |  |  |
| 39 | relay-ballon behind the back | B6 | It is played in pairs. Two people will stand facing their back to each other with a balloon/ ball balanced between their backs and they have to walk/run sideways and reach the therapist without letting the balloon/ ball drop down to the floor. |  |  |  |  |  |  |  |
| 40 | relay-hops and jumps | B7 | Children are divided into even number of teams (2 or 4) and are made to stand in a straight line on the opposite ends of the room. Then, they have to hold a ball in hand and do single leg hops and reach the centre of the room (a cone can be placed). Then, they have to turn 90 degrees (to face the therapist who is standing against the wall) and jump (with both legs) and reach the therapists and after that run back to their team so the next member can take the ball and start hopping. |  |  |  |  |  |  |  |
| 41 | scavenger hunt | B8 | Therapist will name an object that is available in the surroundings and all the children have to go around and find the object. Whoever gets it first, gets a point. |  |  |  |  |  |  |  |
| 42 | walking in lines | B9 | Walking in straight lines: relay of walking forward/ backward/ sideways depending on the command given by the therapist. |  |  |  |  |  |  |  |
| 43 | hopscotch | B10 | Eight boxes will be drawn on the floor in a particular pattern (typical Hopscotch). Children have to jump on one leg across single boxes and both legs across double boxes. They are supposed to complete it without losing balance |  |  |  |  |  |  |  |
| **PA DOMAIN:** **AGILITY** | | |  |  |  |  |  |  |  |  |
| 44 | Dodgeball | A1 | Children form a circle and three children stand inside the circle. The children forming the circle have to throw the ball in such a way that, the ball hits the legs below the knees of any one of the three children inside the circle. The three children inside the circle have to try to dodge the ball by either jumping or moving away quickly. Whoever manages to hit the leg of the person inside the circle must go in next and replace them. |  |  |  |  |  |  |  |
| 45 | dog and the bone | A2 | Children will be divided in two teams. A bone (a cloth piece or an object) will be placed on the floor equidistant from the teams, who are standing opposite to each other. Oneperson from each team comes forward and revolves around the bone. As soon as they get the opportunity, they have to quickly snatch the bone and run towards their team. If the opposite team's member catches him/her with the bone, then they get a point. If the bone is taken back to the team without getting caught, that team gets a point. |  |  |  |  |  |  |  |
| 46 | zig-zag | A3 | Cones will be placed in a straight line, children have to run in a zig zag fashion around the cones |  |  |  |  |  |  |  |
| 47 | t-drills | A4 | A 'T' will be made with cones, children have to walk/ run in forward, lateral and backward direction based on the commands given by the therapist. |  |  |  |  |  |  |  |
| 48 | red light, green light | A5 | Therapist is standing in one corner of the room/ play ground facing away (showing his back) from the children. Children stand in the other corner of the room/ play ground.  Therapist says "red light, green light" several times, and the children run and come and touch the back of the therapist. While doing this, when the therapist stops saying "red light, green light" and turns around and the children have to pause in the place. If someone is found moving they have to go to the start line. |  |  |  |  |  |  |  |
| 49 | prisoners | A6 | Two teams are made on either side of the room. One person from team A comes to team B. team B stands with their arms outstretched. The person from team A claps the hand of any one person from team A and has to run back to his team. If the person who's hand was clapped from team B catches the person from team A that person becomes his prisoner but if the person from team A manages to escape and reach his teammates the person from team B becomes his prisoner. They continue the game, until the last person of either team is left on the same side. |  |  |  |  |  |  |  |
| 50 | colour colour | A7 | There will be one denner (one of the children), who will announce one colour and all kids have to find that colour and touch it.  The denner has the person who hasn't found the colour and he becomes the next denner, or the denner can just sit down and call out 'bubblegum' and all the kids have to run to the denner touch him. The last person to touch the denner becomes the next denner. |  |  |  |  |  |  |  |
| 51 | queen of sheeba | A8 | Children sit in groups. The therapist will say queen of Sheba wants *name of an object* (EG: a hair clip) and one person from each team has to run and find one and give it to the therapist. Whoever gives the item first gets the point. Can be played by dividing into teams as well. |  |  |  |  |  |  |  |
| 52 | simon says | A9 | Therapist says "Simon says" and then commands different activities like "Simon says touch your feet"; "Simon says turn around"; etc., and make them do a variety of activities. |  |  |  |  |  |  |  |
| 53 | balloon up | A10 | Two teams are made and everyone has to try to ensure the balloon does not touch the ground. The teams will take turns to hit the balloon so that it stays off the ground |  |  |  |  |  |  |  |
| 54 | ladders | A11 | Everyone sits in a circle with their legs outstretched and touching each other's legs. The therapist calls out the name of any one child and that child has to get up and jump over everyone's legs and reach the spot he was siting on.  He has to jump/ cross without touching the leg of the children sitting and has to do it as fast as he can |  |  |  |  |  |  |  |
| **ITEM NO.** | **ITEMS** | **ACTIVITY** | **ACTIVITY DESCRIPTION** | **RELEVANCE** | **APPROPRIATENESS** | **CLARITY OF INSTRUCTIONS** | **FEASIBILITY** | **Reviewer Decision** | | |
|  |  |  |  | **Is the activity focusing on the “component of physical activity” it is intended to focus on?** | **Is the activity applicable / performable by children with Down syndrome?** | **Are the instructions clear and simple for children with Down syndrome to follow?** | **Is the activity easy to perform in a special school setting for children with Down syndrome?** | **Accept** | **Accept with modifications** | **Reject** |
|  |  |  |  | **1-5** | **1-5** | **1-5** | **1-5** |  |  |  |
| 55 | musical chairs | A12 | Chairs will be placed in alternative fashion facing opposite directions. The number of chairs will be 2 or 3 less than the number of children (for elimination). The children have to run around the chairs while the music is playing and sit on the chair when music stops, every round 2 or 3 child will get eliminated. 2 or 3 chair will be reduced after each round. |  |  |  |  |  |  |  |
| 56 | musical chairs with numbers | A13 | Chairs will be placed in alternative fashion facing opposite directions, the children have to run around the chairs while the music is playing and sit on the chair when it stops, every round 2-3 children will get eliminated. On each chair there will be a number. the therapist will select a random number using chits and call that out. The person who sits on that chair will have to perform the activity given. |  |  |  |  |  |  |  |
| 57 | crows and cranes | A14 | Two teams will be formed. Both teams will make a line and stand side by side with one foot touching. One team will be named crows, one team will be named cranes. When the therapist says crows, the crows have to run away towards the wall closest to them to be "safe" while the cranes try to catch them before they reach the wall and vice versa |  |  |  |  |  |  |  |
| 58 | kho kho | A15 | Played with 2 teams. One team sits with their backs in opposite directions and space between each other. Two or three people from the opposite team come into the play area and try to escape the catcher from the first team. The catcher is allowed to pat his team mate on the back so that the teammate now becomes the catcher and the previous catcher sits in his or her place. The first team is not allowed to go between their teammates sitting down or change the direction they run in. the second team is allowed to move between the players on the first team and change directions. |  |  |  |  |  |  |  |
| **PA DOMAIN: FLEXIBILITY** | | |  |  |  |  |  |  |  |  |
| 59 | mountain and valleys | F1 | Stand in a line one behind the other, one person lies on the floor like a ball (flexed position) and one person stands with their legs spread apart. The last person jumps over the curled up person and crawls under the spread legs to reach the front of the line and either curls up or spreads his legs and stands (alternate curling up on the floor and standing with legs spread apart) |  |  |  |  |  |  |  |
| 60 | hula hoops | F2 | Make them stand in a circle. Then each child has to pass through the hula hoop and then pass it to the next person and the this continues until everybody in the circle has completed a round. |  |  |  |  |  |  |  |
| 61 | copy me | F3 | One person will demonstrate yoga poses in front of the children and all of them have to try to copy the pose and maintain it for at least 30 seconds |  |  |  |  |  |  |  |
| 62 | limbo | F4 | A stick will be placed. (can be placed at different levels). Children have to pass under the stick and come on the other side. |  |  |  |  |  |  |  |

**S5**. Summary of Expert Responses to Validation Questionnaire in three rounds of Modified Delphi consensus process

| **DOMAINS / ITEMS** | **ACTIVITY** | **Activity Description** | **ROUND 1** | | | | | | | | | | | | |
| --- | --- | --- | --- | --- | --- | --- | --- | --- | --- | --- | --- | --- | --- | --- | --- |
|  |  |  | **Criteria** | | | | **CVI** | | | | **Expert Feedback / Comments / Suggestions** | **Reviewer Decision** | | | **Investigators Remarks** |
|  |  |  | Relevance | Appropriateness | Clarity of Instructions | Feasibility | Relevance | Appropriateness | Clarity of Instructions | Feasibility |  | Accept | Accept with modifications | Reject |  |
| STRENGTH |  |  |  |  |  |  |  |  |  |  |  |  |  |  |  |
| Monkey in the middle | S1 | One person stands in the middle of a circle (monkey). The other children form a circle and they have to throw the ball to each other and not allow the person in the middle to catch the ball. If the monkey catches the ball then the person who threw the ball becomes the next monkey. | 4 | 5 | 6 | 7 | 0.4 | 0.5 | 0.5 | 0.6 | Child is MCI, when the instructions are in series child might underperform, in case of retaining the activity , may consider adding weighted ball to facilitate the intended component; How this will have impacted on strength; This activity seems more relevant to coordination and endurance; Would be nice to include number of students involved in the group and frequency. Simplify the instructions. smaller the group is better. Since you are working on strength would be nice to define the type of ball , because that provide a resistance; As children with trisomy 21 has low muscle tone they will have a delay in their milestones and coordination and balance ; Activity doesn’t focus on strength; | 4 | 7 | 0 | Next Round |
| Kicks | S2 | Kick the ball into the goal post, either facing the goal post or facing to the side and kicking sideways into the goal post. Each child gets 5 or 10 chances. Total goals are counted. | 10 | 9 | 10 | 9 | 0.9 | 0.8 | 0.9 | 0.8 | Easy to understand and perform; because its sideways, understanding will be a problem; This may not come under strength; easy and straightforward activity; doable; Physical therapist should assess the muscle tone and give appropriate strengthening ex’s so that the child can perform the above activities; | 10 | 1 | 0 | Accept |
| Frog Jump | S3 | Children have to be in full squat with hands on the ground, they have to push on the ground with all four limbs and jump to go ahead. They have to to do this from starting to ending point (like a race) | 9 | 8 | 8 | 8 | 0.8 | 0.7 | 0.7 | 0.7 | Fun activity; Difficult??; Avoid the child from jumping activities they have potential for atlanto axial joint dislocation; easy and fun activity appropriate for testing explosive strength in lower limbs; would be nice to modify the instructions; Great activity but children with moderate cognitive impairment might not understand how to do the jump; | 3 | 8 | 0 | Next Round |
| Rabbit Jump | S4 | Children have to half squat and jump. They must do this from starting to ending point (like a race) | 8 | 8 | 8 | 6 | 0.7 | 0.7 | 0.7 | 0.5 | Child may perform frog jump itslef; How strength? to flexible structure they would do it easily; to maintain half squat might be difficult; avoid jumping; quite similar to the previous activity; doable | 1 | 5 | 5 | Next Round |
| Military crawl | S5 | Children have to crawl on forearms while dragging their lower body forwards. They must do this from starting to ending point (like a race) | 11 | 9 | 11 | 10 | 1.0 | 0.8 | 1.0 | 0.9 | Challenging activity; may require an area which is carpetted or has mats; description of the activity could be modified | 9 | 2 | 0 | Accept |
| Catch & throw | S6 | Children stand in a circle. The children catch and throw a ball to each other/ to the therapist. (Therapist can stand in the centre of the circle) | 7 | 7 | 10 | 10 | 0.6 | 0.6 | 0.9 | 0.9 | same as the first activity; repeated activity (similar); Not sure whether it will come under strength domain; This activity also will require good coordination; same activity as the monkey in the middle. more so it is endurance purpose | 2 | 7 | 2 | Next Round |
| Target | S7 | There are two teams who are standing opposite to each other at a distance. There is mark on the floor halfway between the starting point of both the teams, where a bucket is placed. The person standing in the first aims and tries to throw the ball into the bucket as many times as possible for 10 seconds and runs back and joins the line. The next child in line continues the same. | 8 | 8 | 7 | 8 | 0.7 | 0.7 | 0.6 | 0.7 | May be difficult to make them understand; Not sure whether it will come under strength domain; Not sure how the teams are standing, will the children be patient enough till its their turn; Again the same query, this is strength component , focusing on that component would be great. throwing as many as times - endurance ? | 5 | 6 | 0 | Next Round |
| Arm Wrestling | S8 | Two individuals sit across the table and hold the other person's hand with their elbows on the table. They try to push each other's hand down, to the side to touch the table. | 10 | 9 | 11 | 10 | 0.9 | 0.8 | 1.0 | 0.9 | This can be done in apparently normal children as it may be discouraging to the opponent who will loose; may not be apprpriate; Easy activity to understand; doable | 9 | 1 | 1 | Accept |
| Leg Wrestling | S9 | Two people lie on the mat and intwine one of their legs. On the count of three, each one has to try to push the other person's leg down and make it touch the ground. Whoever makes the other person's leg touch the floor, wins the game. | 6 | 4 | 4 | 6 | 0.5 | 0.4 | 0.4 | 0.5 | Risk of injury; The state of joint laxity must be considered with high intensity activity; Not clear whether the children have to lie next to each other or opposite to each other; not sure about children following the instructions. the activity seems to be slightly challenging; | 0 | 6 | 5 | Next Round |
| Duck Walk | S10 | Children do a complete squat and then have to walk while squatting. They must do this from starting to ending point (like a race) | 8 | 9 | 8 | 7 | 0.7 | 0.8 | 0.7 | 0.6 | balancing may be a challenge; concern with respect to hypermobility , feet is already in pronation . in addition understanding the activity is challenging; | 5 | 6 | 0 | Next Round |
|  |  |  |  |  |  |  |  |  |  |  |  |  |  |  |  |
| ENDURANCE |  |  |  |  |  |  |  |  |  |  |  |  |  |  |  |
| Blind man's bluff | E1 | One person (teacher/ therapist) blind folds themselves and attempts to catch the children while they try to run around and avoid getting caught by the blindfolded person. Another person (teacher/therapist) can take care of the safety of the children and the blindfolded person | 7 | 6 | 7 | 8 | 0.6 | 0.5 | 0.6 | 0.7 | Risk of fall; The temporal factor must be considered; Needs to add durations; safety?; As the child is MR.on top of that you wil blind fold, its harmful for the child; Blind folding; increases the level of difficulty, could consider playing chain instead; Concern regarding the safety aspect. | 3 | 8 | 0 | Next Round |
| fire in the mountain | E2 | Children run in a circle (holding hands) singing "fire in the mountain, run, run, run" while the person in the middle shouts a random number, the children have to break the circle and form groups of that specific number that was shouted. Whoever could not form the specific group are considered 'out'. The game continues by forming the circle again. Game is played until last one/two children are left. | 11 | 11 | 10 | 9 | 1.0 | 1.0 | 0.9 | 0.8 | forming groups based on number, help might be required; children may require a demo to understand the game but its doable; there is cognitive task also involved. Would be nice to focus one component. | 9 | 2 | 0 | Accept |
| reverse dodgeball | E3 | One person (therapist/teacher) stand in the middle of the circle, the other children form a circle around and stand at a distance. the person in the middle who has to throw the ball below the knee level on the children forming the circle and get them out of the game. The children forming the circle have to dodge the ball either by jumping or moving away | 5 | 5 | 5 | 5 | 0.5 | 0.5 | 0.5 | 0.5 | not applicable; repeated, similar; please refer evidence; Fun activity but again requires supervision for safety; how does this would be involved in endurance | 2 | 9 | 0 | Next Round |
| sea and shore | E4 | One side of the room/ ground is designated as sea, the opposite side as shore. The person conducting the games gives command: 'sea' (run to the side designated as sea); 'shore' (run to the side designated as shore); 'rains falling' (squat and sit with hands on the head); 'captains calling' (stand and salute); 'freeze' (freeze in whatever position you currently are).  Whoever makes a mistake in following the commands is out of the game and has to sit on the sides. Continue until last few children (4-5) are left | 11 | 9 | 7 | 7 | 1.0 | 0.8 | 0.6 | 0.6 | multiple commands may not be feasible; multiple commnads, might be difficult for the children to comprehend; No sea shore activities to avoid jerk in atlantoaxial joints; There are too many commands. Restrict the commands to any two for a while, then do; Very complex activity , not sure about the understanding of this activity; Might be difficult for the children who have moderate cognitive impairments | 5 | 6 | 0 | Next Round |
| football | E5 | Children will be divided into two groups. The aim of the game is to aim and kick the ball into the goalpost (giving one goal/ point to that team). The teams will compete to run and get the ball and kick it onto the goalpost to make a goal | 11 | 11 | 9 | 10 | 1.0 | 1.0 | 0.8 | 0.9 | Provided timed and increased progressively; similar, repeated; precaution to be taken; will the whole team run to get the ball or one person from each team?; Doable | 10 | 1 | 0 | Accept |
| basketball | E6 | Children will be divided into two groups, the children stand in a line and one after the other. They have to dribble the ball 3 times and then have to aim the ball and deliver it into the basket (typical basketball basket). Each child will get 3 chances. They compete for whichever team puts more baskets. | 10 | 10 | 10 | 8 | 0.9 | 0.9 | 0.9 | 0.7 | Provided timed and increased progressively; Dribbling may be difficult; avoid jumping activities; Straightforward activity, may need to modify the height of the basket to make it easier doable | 8 | 3 | 0 | Next Round |
| tag | E7 | There will be a denner (one of the children) who will run and catch the other children. The other children will run in the given space without getting caught. If they are touched by the denner then they are considered out and they become the next denner. | 11 | 11 | 11 | 11 | 1.0 | 1.0 | 1.0 | 1.0 | Easy; with precaution; Easy and appropriate; doable | 11 | 0 | 0 | Accept |
| cow and tiger | E8 | All children will be made to stand in a circle while holding hands, two children will be selected of which one will be a tiger and one will be a cow. The tiger will to be outside the circle and cow will be inside the circle. The cow has to run away from the tiger and can freely go under the hands of the children forming the circle. But the tiger needs permission (from the children forming the circle) to go inside or outside. When he gets the permission, the tiger runs and tries to catch the cow.  If the cow gets caught then the therapist decides who will be the tiger and the cow for the next round. If the cow does not get caught then the therapists changes the cow and the tiger after 1-2mins of running | 10 | 9 | 8 | 8 | 0.9 | 0.8 | 0.7 | 0.7 | very complex; with precaution and command; May be complicated to understand and do; doable | 6 | 5 | 0 | Next Round |
| single leg hops | E9 | One child will be the denner. The denner has to hop on single leg and catch the other players who are running in the given space without getting caught. The child who gets caught is the next denner. | 8 | 8 | 8 | 7 | 0.7 | 0.7 | 0.7 | 0.6 | More of balance task; balance and safety?; no hopping activities; May be quite difficult for a child with DS to hop on one leg for long; I am afraid the children will be able to run on the single leg | 4 | 7 | 0 | Next Round |
| lock and key | E10 | There will be one denner. All other children are running in a given space. If the denner touches any player, they will get 'lock'ed and that player has to stand in the same spot and cannot run.  The other players can release the ones who are 'lock'ed by touching them and giving them a 'key', then they can start running again. The children have to try to run around and escape from getting 'lock'ed. If a child who is 'lock'ed takes a step/runs without getting a 'key'; he becomes a denner. | 10 | 9 | 9 | 9 | 0.9 | 0.8 | 0.8 | 0.8 | Understanding may be the key here; Instructions are clear but requires a lot of team coordination and quick reaction so not sure if eaveryone in the group would understand; Doable | 9 | 2 | 0 | Accept |
| chain cut | E11 | There will be a single denner at the beginning of the game, who will start by catching one player. Then, both of them will hold hands and catch another player at random. Each time one person is caught, they will hold hands of the previously caught players and catch the next person. The chain continues to grow till the last person is caught. The chain cannot break while running, if it does then the two people who broke the chain start from the beginning and catch people to increase the chain. | 10 | 10 | 10 | 9 | 0.9 | 0.9 | 0.9 | 0.8 | can be done; Appropriate and easy though it requires team work and supervision for safety; safety concerns, as they are running holding hands | 10 | 1 | 0 | Accept |
| run in a circle | E12 | All children will stand in a circle. The therapist will give the following instructions- 'If I blow the whistle once you have to run clockwise, if I blow the whistle twice you have to run anticlockwise and if I blow the whistle thrice you stand still'.  Whoever makes a mistake in following the command, gets out of the game and sits. | 9 | 7 | 9 | 8 | 0.8 | 0.6 | 0.8 | 0.7 | commands could be complex; can be done; Will cause lot of confusion and children's safety may be an issue as they may bang into each other and fall; this sounds like cognitive task | 8 | 3 | 0 | Next Round |
| cut the cake | E13 | Everyone stands in a circle holding hands. One pair runs outside the circle and touches the pair of hands they want to challenge. The two people whose interlocked hands have been touched have to run. | 6 | 6 | 7 | 6 | 0.5 | 0.5 | 0.6 | 0.5 | Complicated; consider the child has less IQ; Not clear about the instructions and whether children with DS will be able to follow; doable | 5 | 6 | 0 | Next Round |
| cricket | E14 | Divide them into 2 groups and play the traditional game of cricket. (or) Give each child a chance to bat for 5 balls and then the other children can take chances at throwing the ball or doing fielding. | 10 | 9 | 10 | 10 | 0.9 | 0.8 | 0.9 | 0.9 | consider their joint stability; May be complicated as it requires team work with different expectations; doable | 10 | 1 | 0 | Accept |
|  |  |  |  |  |  |  |  |  |  |  |  |  |  |  |  |
| CO-ORDINATION |  |  |  |  |  |  |  |  |  |  |  |  |  |  |  |
| We move | C1 | Everyone stands in a circle. The song is sung and they have to follow the following commands and move accordingly: 'we move to the left' (side shuffle two spaces to the left)  'we move to the right' (side shuffle two spaces to the right) 'we move and move' (side shuffle two places to the left)  'move all night' (side shuffle two places to the right) 'heel and toe' (point heel and toe alternately)  'half way go' (turn halfway)  'a new friend is here' (spin/ turn around and point to the person standing next to them)" | 10 | 9 | 8 | 8 | 0.9 | 0.8 | 0.7 | 0.7 | complex; groups have to be divided properly considering the IQ level; Too complicated; spell check ? move all night | 8 | 3 | 0 | Next Round |
| head, shoulders, knee | C2 | Two people stand on either sides of a cone. They have to touch their head, shoulders or knees as per the commands given by the person conducting the game (therapist/teacher). When the person conducting gives the command 'catch' they have to quickly grab the cone. The first to grab the cone gets the point | 11 | 10 | 9 | 8 | 1.0 | 0.9 | 0.8 | 0.7 | Command may be difficult to understand; understandability of commands?; can be done; Instructions may be confusing | 5 | 6 | 0 | Next Round |
| passing the parcel | C3 | Everyone stands in a circle, music is played. The therapist gives them an object to be passed around while playing music. Once the music stops the person with the object in hand has to perform a task (Example:10 squats, 5 lunges, dance for 30 seconds, 7 jumps, etc.,). | 8 | 10 | 10 | 10 | 0.7 | 0.9 | 0.9 | 0.9 | remove jumping; Does not require too much coordination? | 8 | 3 | 0 | Next Round |
| big fish small fish | C4 | The therapist will give commands of "big fish" where the children have to hold their arms out at a wide distance to show the size of the fish, and "small fish" where they will reduce the distance between their hands to depict a small fish. The commands get faster with time. | 11 | 10 | 9 | 9 | 1.0 | 0.9 | 0.8 | 0.8 | activity is command; not relevant consider the IQ of a child; Doable; doable | 10 | 1 | 0 | Accept |
| three legged race | C5 | It is a race to be done in pairs. One person will tie their left leg to the right leg of the other person in the team and they both have to coordinate their leg movements and run to the finish line. | 8 | 6 | 7 | 5 | 0.7 | 0.5 | 0.6 | 0.5 | Risk of fall; Difficult and not safe; safety issues involved; May be a concern for safety; safety concerns | 0 | 6 | 5 | Next Round |
| hidden items | C6 | One person will run from each team to the therapist who will show them a picture which can be any sort of puzzle such as find the difference/find the hidden item/find the hidden word. Each person finds and circles one item and runs back to their team. First team to find all the items wins. | 6 | 6 | 6 | 6 | 0.5 | 0.5 | 0.5 | 0.5 | not relevant for coordination; Seems more like a cognitive activity; what component of coordination is involved ?; not applicable | 0 | 6 | 5 | Next Round |
| gongee | C7 | Children will be given five stones, he has to first throw the stones on the ground. He has to stand with one leg behind the other (Tandem standing) and pick up the stones. If the stones are apart, he can walk with one leg behind the other (tandem walking) and pick them. | 7 | 7 | 5 | 7 | 0.6 | 0.6 | 0.5 | 0.6 | This is more of balance task; Difficult to understand; complex; can be done; seems more relevant to balance; instructions are not clear | 3 | 8 | 0 | Next Round |
| message receieved | C8 | Everyone sits in a circle holding hands (hook grasp) with the denner (Child or therapist) in the middle. The game starts from one person in the circle who says I want to pass my message to 'x' (name of any child).  After saying that, the person gently presses the hand of the person sitting on any one side (he/she can press the hands of one person only, either left or right side). That person, then passes the message forward by gentling pressing the hand of the person next to him, This continues such that the message reaches 'x'. Once 'x' receives the message he has to say 'message received'.  The denner has to observe the hand movements while the message travels from the sender to the recipient and if he observes any hand movement then he has to call out their name and they become the next denner. | 6 | 6 | 7 | 7 | 0.5 | 0.5 | 0.6 | 0.6 | can be done; Not related to coordination and again maybe difficult to perform as a team; what component of coordination is involved ? | 0 | 7 | 4 | Next Round |
| dribble the ball | C9 | Child has to dribble the ball on the floor and run from one end of the room to another | 10 | 9 | 10 | 10 | 0.9 | 0.8 | 0.9 | 0.9 | even includes agility; can be under endurance; Can just do dribbling and count the number of time each child can dribble.. running with dribbling may be too challenging; doable | 10 | 1 | 0 | Accept |
|  |  |  |  |  |  |  |  |  |  |  |  |  |  |  |  |
| BALANCE |  |  |  |  |  |  |  |  |  |  |  |  |  |  |  |
| side shuffles | B1 | Two people stand opposite each other throwing and catching a ball while walking side ways | 10 | 10 | 10 | 9 | 0.9 | 0.9 | 0.9 | 0.8 | may also add to eye hand coordination apart from balance; difficult for healthy children itself; seems doable; doable | 10 | 1 | 0 | Accept |
| crocodile can I cross the river | B2 | The denner is the therapist. The players have to ask the denner if they can cross the river, if he says yes then they jump forward, if he says no then they have to jump back once | 10 | 8 | 7 | 10 | 0.9 | 0.7 | 0.6 | 0.9 | avoid jumping; Where do the children need to reach? May cause confusion while jumping and they might just copy each other more than processing the command and acting by themselves | 7 | 4 | 0 | Next Round |
| in the pond, on the bank | B3 | A line will be drawn on the floor. The therapist will give commands of "in the pond" then the children jump forward or "on the bank" the stay on this side of the river/ jump back (in case they are on the other side of the pond) | 8 | 9 | 8 | 9 | 0.7 | 0.8 | 0.7 | 0.8 | complex commands; Repetition of previous; would it be better to have an elevated platform from which they just step down or step up rather than jump; what is the difference from the previous activity ? | 3 | 8 | 0 | Next Round |
| relay-book balancing | B4 | Children are divided into teams. (3 to 5 teams depending on the total number of children). It is a race, where the child has to balance a book on the head (should not touch/hold the book with hands) and reach the therapist and then hold the book in his hand and run back to the team. Then the next member in the team can repeat the same. Team that finishes the relay first, wins. | 9 | 10 | 11 | 9 | 0.8 | 0.9 | 1.0 | 0.8 | Give proper instruction; The first component of balancing the book itself is enough for balance activity; doable | 11 | 0 | 0 | Accept |
| relay-lemon and spoon | B5 | Place the behind part of a spoon in your mouth, place a lemon on the spoon and race to a finish line. It can be done in slots (7-8 children per slot) and winners can be identified for each slot. | 11 | 10 | 11 | 10 | 1.0 | 0.9 | 1.0 | 0.9 | with proper command; Good activity for promoting mouth closure too; doable | 11 | 0 | 0 | Accept |
| relay-ballon behind the back | B6 | It is played in pairs. Two people will stand facing their back to each other with a balloon/ ball balanced between their backs and they have to walk/run sideways and reach the therapist without letting the balloon/ ball drop down to the floor. | 10 | 9 | 10 | 10 | 0.9 | 0.8 | 0.9 | 0.9 | Not appropriate; chances of falling are high; Doable; doable | 8 | 3 | 0 | Accept |
| relay-hops and jumps | B7 | Children are divided into even number of teams (2 or 4) and are made to stand in a straight line on the opposite ends of the room. Then, they have to hold a ball in hand and do single leg hops and reach the centre of the room (a cone can be placed). Then, they have to turn 90 degrees (to face the therapist who is standing against the wall) and jump (with both legs) and reach the therapists and after that run back to their team so the next member can take the ball and start hopping. | 8 | 8 | 7 | 6 | 0.7 | 0.7 | 0.6 | 0.5 | Multiple tasks incorporated, simplify; complex; It should not be praticed; Too complicated and has components of strength and endurance; doable | 0 | 7 | 4 | Next Round |
| scavenger hunt | B8 | Therapist will name an object that is available in the surroundings and all the children have to go around and find the object. Whoever gets it first, gets a point. | 6 | 6 | 7 | 6 | 0.5 | 0.5 | 0.6 | 0.5 | Not applicable; its not relevant under balance; Not sure how it is related to balance and again may cause too much chaos; what component of balance is trained here ? | 0 | 6 | 5 | Next Round |
| walking in lines | B9 | Walking in straight lines: relay of walking forward/ backward/ sideways depending on the command given by the therapist. | 11 | 11 | 11 | 11 | 1.0 | 1.0 | 1.0 | 1.0 | Easy task; Tandem walking you can write; Doable; doable | 11 | 0 | 0 | Accept |
| hopscotch | B10 | Eight boxes will be drawn on the floor in a particular pattern (typical Hopscotch). Children have to jump on one leg across single boxes and both legs across double boxes. They are supposed to complete it without losing balance | 9 | 8 | 8 | 8 | 0.8 | 0.7 | 0.7 | 0.7 | incorporates the coordinaton component as well; please avoid jumping; May be slightly difficult for some | 6 | 5 | 0 | Next Round |
|  |  |  |  |  |  |  |  |  |  |  |  |  |  |  |  |
| AGILITY |  |  |  |  |  |  |  |  |  |  |  |  |  |  |  |
| Dodgeball | A1 | Children form a circle and three children stand inside the circle. The children forming the circle have to throw the ball in such a way that, the ball hits the legs below the knees of any one of the three children inside the circle. The three children inside the circle have to try to dodge the ball by either jumping or moving away quickly. Whoever manages to hit the leg of the person inside the circle must go in next and replace them. | 9 | 9 | 10 | 10 | 0.8 | 0.8 | 0.9 | 0.9 | repeated; what if the child fall; doable with supervision; doable | 10 | 1 | 0 | Accept |
| dog and the bone | A2 | Children will be divided in two teams. A bone (a cloth piece or an object) will be placed on the floor equidistant from the teams, who are standing opposite to each other. Oneperson from each team comes forward and revolves around the bone. As soon as they get the opportunity, they have to quickly snatch the bone and run towards their team. If the opposite team's member catches him/her with the bone, then they get a point. If the bone is taken back to the team without getting caught, that team gets a point. | 10 | 10 | 10 | 10 | 0.9 | 0.9 | 0.9 | 0.9 | Generally we do not need agility if not a sports person. The exercises mentioned are for healthy children that too those who are athletes. Needs to understand the condition well and read more articles to form exercises, no where swimming cycling are mentioned. | 10 | 1 | 0 | Accept |
| zig-zag | A3 | Cones will be placed in a straight line, children have to run in a zig zag fashion around the cones | 11 | 11 | 11 | 11 | 1.0 | 1.0 | 1.0 | 1.0 | with precaution | 11 | 0 | 0 | Accept |
| t-drills | A4 | A 'T' will be made with cones, children have to walk/ run in forward, lateral and backward direction based on the commands given by the therapist. | 10 | 10 | 10 | 9 | 0.9 | 0.9 | 0.9 | 0.8 | can the child follow the command? they have less IQ; Doable if done individually; doable | 10 | 1 | 0 | Accept |
| red light, green light | A5 | Therapist is standing in one corner of the room/ play ground facing away (showing his back) from the children. Children stand in the other corner of the room/ play ground.  Therapist says "red light, green light" several times, and the children run and come and touch the back of the therapist. While doing this, when the therapist stops saying "red light, green light" and turns around and the children have to pause in the place. If someone is found moving they have to go to the start line. | 7 | 7 | 7 | 7 | 0.6 | 0.6 | 0.6 | 0.6 | complex commands; please do not confuse the child as they have less IQ and they probably ending up hurting themselves | 3 | 8 | 0 | Next Round |
| prisoners | A6 | Two teams are made on either side of the room. One person from team A comes to team B. team B stands with their arms outstretched. The person from team A claps the hand of any one person from team A and has to run back to his team. If the person who's hand was clapped from team B catches the person from team A that person becomes his prisoner but if the person from team A manages to escape and reach his teammates the person from team B becomes his prisoner. They continue the game, until the last person of either team is left on the same side. | 8 | 6 | 8 | 7 | 0.7 | 0.5 | 0.7 | 0.6 | its not agility; May be challenging | 3 | 8 | 0 | Next Round |
| colour colour | A7 | There will be one denner (one of the children), who will announce one colour and all kids have to find that colour and touch it.  The denner has the person who hasn't found the colour and he becomes the next denner, or the denner can just sit down and call out 'bubblegum' and all the kids have to run to the denner touch him. The last person to touch the denner becomes the next denner. | 7 | 7 | 8 | 7 | 0.6 | 0.6 | 0.7 | 0.6 | consider the IQ | 3 | 8 | 0 | Next Round |
| queen of sheeba | A8 | Children sit in groups. The therapist will say queen of Sheba wants *name of an object* (EG: a hair clip) and one person from each team has to run and find one and give it to the therapist. Whoever gives the item first gets the point. Can be played by dividing into teams as well. | 9 | 9 | 10 | 8 | 0.8 | 0.8 | 0.9 | 0.7 | Not relevant for agility | 1 | 10 | 0 | Next Round |
| simon says | A9 | Therapist says "Simon says" and then commands different activities like "Simon says touch your feet"; "Simon says turn around"; etc., and make them do a variety of activities. | 11 | 11 | 11 | 11 | 1.0 | 1.0 | 1.0 | 1.0 | Similar activity as previous; Not sure how this is an agility activity; doable | 11 | 0 | 0 | Accept |
| balloon up | A10 | Two teams are made and everyone has to try to ensure the balloon does not touch the ground. The teams will take turns to hit the balloon so that it stays off the ground | 8 | 8 | 7 | 8 | 0.7 | 0.7 | 0.6 | 0.7 | even included eye hand coordination; Its not agility; may have safety concerns if they bang into each other, what is the purpose of two teams | 2 | 9 | 0 | Next Round |
| ladders | A11 | Everyone sits in a circle with their legs outstretched and touching each other's legs. The therapist calls out the name of any one child and that child has to get up and jump over everyone's legs and reach the spot he was siting on.  He has to jump/ cross without touching the leg of the children sitting and has to do it as fast as he can | 9 | 8 | 8 | 7 | 0.8 | 0.7 | 0.7 | 0.6 | safety? child might stamp other's feet; its not agility and child should not jump; May be difficult; safety concern; Safety? | 1 | 9 | 1 | Next Round |
| musical chairs | A12 | Chairs will be placed in alternative fashion facing opposite directions. The number of chairs will be 2 or 3 less than the number of children (for elimination). The children have to run around the chairs while the music is playing and sit on the chair when music stops, every round 2 or 3 child will get eliminated. 2 or 3 chair will be reduced after each round. | 10 | 10 | 10 | 10 | 0.9 | 0.9 | 0.9 | 0.9 | its not relevant for agility | 10 | 1 | 0 | Accept |
| musical chairs with numbers | A13 | Chairs will be placed in alternative fashion facing opposite directions, the children have to run around the chairs while the music is playing and sit on the chair when it stops, every round 2-3 children will get eliminated. On each chair there will be a number. the therapist will select a random number using chits and call that out. The person who sits on that chair will have to perform the activity given. | 9 | 9 | 8 | 9 | 0.8 | 0.8 | 0.7 | 0.8 | Similar as previous item; complex; its not relevant; In terms of agility, not different from the previous one | 1 | 10 | 0 | Next Round |
| crows and cranes | A14 | Two teams will be formed. Both teams will make a line and stand side by side with one foot touching. One team will be named crows, one team will be named cranes. When the therapist says crows, the crows have to run away towards the wall closest to them to be "safe" while the cranes try to catch them before they reach the wall and vice versa | 9 | 8 | 8 | 8 | 0.8 | 0.7 | 0.7 | 0.7 | complex commands; not relevant; instructions are not clear | 2 | 9 | 0 | Next Round |
| kho kho | A15 | Played with 2 teams. One team sits with their backs in opposite directions and space between each other. Two or three people from the opposite team come into the play area and try to escape the catcher from the first team. The catcher is allowed to pat his team mate on the back so that the teammate now becomes the catcher and the previous catcher sits in his or her place. The first team is not allowed to go between their teammates sitting down or change the direction they run in. the second team is allowed to move between the players on the first team and change directions. | 9 | 7 | 8 | 6 | 0.8 | 0.6 | 0.7 | 0.5 | even includes endurance component; understanding the rules?; child must have less IQ; May be very challenging; doable | 2 | 9 | 0 | Next Round |
|  |  |  |  |  |  |  |  |  |  |  |  |  |  |  |  |
| FLEXIBILITY |  |  |  |  |  |  |  |  |  |  |  |  |  |  |  |
| mountain and valleys | F1 | Stand in a line one behind the other, one person lies on the floor like a ball (flexed position) and one person stands with their legs spread apart. The last person jumps over the curled up person and crawls under the spread legs to reach the front of the line and either curls up or spreads his legs and stands (alternate curling up on the floor and standing with legs spread apart) | 8 | 6 | 6 | 5 | 0.7 | 0.5 | 0.5 | 0.5 | safety; not relevant for hildren with downs; too complicated and may not be safe; doable | 4 | 7 | 0 | Next Round |
| hula hoops | F2 | Make them stand in a circle. Then each child has to pass through the hula hoop and then pass it to the next person and the this continues until everybody in the circle has completed a round. | 8 | 7 | 9 | 9 | 0.7 | 0.6 | 0.8 | 0.8 | what component of flexibility trained? | 3 | 8 | 0 | Next Round |
| copy me | F3 | One person will demonstrate yoga poses in front of the children and all of them have to try to copy the pose and maintain it for at least 30 seconds | 11 | 10 | 11 | 10 | 1.0 | 0.9 | 1.0 | 0.9 | Avoid stretching of neck flexors and extensors other wise its a good exercise | 11 | 0 | 0 | Accept |
| limbo | F4 | A stick will be placed. (can be placed at different levels). Children have to pass under the stick and come on the other side. | 10 | 10 | 11 | 11 | 0.9 | 0.9 | 1.0 | 1.0 | safety; Kind off same as previous; Doable; doable | 10 | 1 | 0 | Accept |

| **DOMAINS / ITEMS** | **ACTIVITY** | **Modified Activity Description /**  **Query to Panel Experts** | **ROUND 2** | | | | | | | | | | | | |
| --- | --- | --- | --- | --- | --- | --- | --- | --- | --- | --- | --- | --- | --- | --- | --- |
|  |  |  | **Criteria** | | | | **CVI** | | | | **Expert Feedback / Comments / Suggestions** | **Reviewer Decision** | | | **Investigators Remarks** |
|  |  |  | Relevance | Appropriateness | Clarity of Instructions | Feasibility | Relevance | Appropriateness | Clarity of Instructions | Feasibility |  | Accept | Accept with modifications | Reject |  |
| STRENGTH |  |  |  |  |  |  |  |  |  |  |  |  |  |  |  |
| Monkey in the middle | S1 | One person (monkey) stands in the middle of a circle formed by children (group of 6 to 8 children can form the circle. The other children have to throw the ball to each other and not allow the person in the middle (monkey) to catch the ball (use a ball which is around 0.5 kg/ a basketball). If the monkey catches the ball then the person who threw the ball becomes the next monkey/comes to the middle. | 10 | 10 | 10 | 10 | 0.9 | 0.9 | 0.9 | 0.9 | May be used for strength training; What factors will be considered for adding resistance? How much resistance?; Appropriate; doable; Adopt the modified activity; adding weight to the ball is good idea; | 9 | 2 | 0 | Accept |
| Frog Jump | S3 | Therapist can demonstrate the Frog jump and children can try to copy and gradually over the sessions learn to perform the jump. | 11 | 11 | 11 | 11 | 1.0 | 1.0 | 1.0 | 1.0 | Maybe retained with modification; Appropriate; retain; Feet is hypermobile, Physio need to consider this point while exerciseing . Prior in detail assement is very important to rule out any laxity at ankle joint; modification - demo is good idea but many children will find it difficult; | 9 | 2 | 0 | Accept |
| Rabbit Jump | S4 | Any modifications that the expert would like to suggest? | 7 | 7 | 8 | 5 | 0.6 | 0.6 | 0.7 | 0.5 | May be eliminated as it is a repetition and considering the difficulty | 1 | 4 | 6 | Reject |
| Catch & throw | S6 | Any modifications that the expert would like to suggest? | 6 | 7 | 7 | 7 | 0.5 | 0.6 | 0.6 | 0.6 | May be eliminated as it is a repetition; Repetition of activity. Keep any 1. Better eliminate this item; Appropriate; this activity may not be under strength heading; Eliminate; eliminate; same activity as the monkey in the middle; retain any one of the two; Same as the monkey activity. Could eliminate or modify- Jump squat and throw ball?; similar to monkey in the middle; | 1 | 4 | 6 | Reject |
| Target | S7 | If the activity can be shifted to co-ordination | 9 | 9 | 10 | 9 | 0.8 | 0.8 | 0.9 | 0.8 | May be used to improve the coordination; Can be put under Endurance; Appropriate; can be shifted to coordination domain; eliminate the activity. not required to shift to the coordination domain, as that domain already consists of 6 exercise; this is both coordination and strength; Yes, can be shifted to coordination domain; different coloured circle for each can be made for coming back and standing in the line; | 4 | 7 | 0 | Shift to Coordination & Accept |
| Leg Wrestling | S9 | Two people lie on the mat opposite to each other and intwine one of their legs. On the count of three, each one has to try to push the other person's leg down to touch the ground. Whoever makes the other person's leg touch the floor, wins the game. Any modifications that the expert would like to suggest? Retain/ eliminate the activity- please comment | 6 | 6 | 6 | 6 | 0.5 | 0.5 | 0.5 | 0.5 | May be used with precaution due to the risk of injury; Can be eliminated; Appropriate; Eliminate; Eliminate; Eliminate , as mentioned earlier cognition and tone both are concern; Retain; In addition, extremely close proximity of children may be uncomfortable; might kick each other; | 0 | 4 | 7 | Reject |
| Duck Walk | S10 | Any modifications that the expert would like to suggest? | 7 | 7 | 7 | 7 | 0.6 | 0.6 | 0.6 | 0.6 | If some support like a pushcart can be provided, it will make the task easier; Retain; Appropriate; retain; Move it up in sequence; eliminate this activity; eliminate- hypermobility is an issue; Avoid complete squat as it pushes the joint in end range. Could do a half squat instead. | 5 | 5 | 1 | Next Round |
| ENDURANCE |  |  |  |  |  |  |  |  |  |  |  |  |  |  |  |
| Blind man's bluff | E1 | Since the children are not blindfolded but a teacher/ therapist is blindfolded and one more therapist is monitoring the entire game- can the game be included? Any modifications that the expert would like to suggest? Retain/ eliminate the activity- please comment | 9 | 9 | 9 | 9 | 0.8 | 0.8 | 0.8 | 0.8 | May be retained with safety precautions; Retain; No change needed; can be retain; Retain; If there is a physiotherapist to monitor the acitivty you could go ahead with the activity; retain; Can be included with precautions - safe area, help available, etc.; eliminate; risky, safety issues | 8 | 3 | 0 | Accept |
| reverse dodgeball | E3 | Any modifications that the expert would like to suggest? | 9 | 9 | 9 | 10 | 0.8 | 0.8 | 0.8 | 0.9 | Activity may be used for older children for coordination; Retain; No change needed; can be shifted to coordination; This activity is more of power component; safety concerns.. eliminate; There is no scope of running in reverse dodge ball. Regular dodge ball may work; children throw hard; | 6 | 4 | 1 | Shift to Coordination & Accept |
| sea and shore | E4 | Modification: Start with only two commands in the beginning and as they get familiarized with them, gradually over the period of a few sessions add the other commands one at a time. | 11 | 11 | 10 | 10 | 1.0 | 1.0 | 0.9 | 0.9 | May be retained with the modification; No change needed; can be retained; relavant; retain with the modification; Fun! You could draw islands in between with chalk too!; understanding complex commands will be difficult; No change needed; can be retained; relavant; retain with the modification; | 9 | 2 | 0 | Accept |
| basketball | E6 | Any modifications that the expert would like to suggest? | 9 | 9 | 11 | 10 | 0.8 | 0.8 | 1.0 | 0.9 | Activity seems to be appropriate for coordination; Retain; No change needed; Instead of dribbling children can go for a certain distance and then aim the ball; Eliminate the dribbling part.. just run with the ball in hands and put through the basket; There is no major jumping here- so its ok. | 8 | 3 | 0 | Accept |
| cow and tiger | E8 | Can the activity be learnt by practicing over a few sessions? | 8 | 7 | 8 | 7 | 0.7 | 0.6 | 0.7 | 0.6 | May be used for older children | 4 | 7 | 0 | Next Round |
| single leg hops | E9 | Modifications: One child will be the denner. The denner has to hop on single leg 3 times in the beginning of the game and then he can run and catch the other players who are running in the given space without getting caught. The child who gets caught is the next denner. | 9 | 9 | 10 | 10 | 0.8 | 0.8 | 0.9 | 0.9 | May be used for both endurance and balance; Since it is single leg hopping better shift to Balance domain; No change needed; can be eliminated; Balance; unsure about following commands of three times hopes and then run. Since running is invloved you can consider this activity for endurance; if child cannot hop then its just a regular run and catch. so just keep it as run and catch; Could be in either domain. Balance seems more appropriate; balance domain; | 2 | 8 | 1 | Shift to Balance & Accept |
| run in a circle | E12 | Modification: Start with only one command in the beginning and as they get familiarized with them, gradually over the period of a few sessions add the other commands one at a time. | 11 | 11 | 10 | 10 | 1.0 | 1.0 | 0.9 | 0.9 | May be retained with the modification; No change needed; can be retained; retain; start with walking instead of running; complex commands; | 10 | 1 | 0 | Accept |
| cut the cake | E13 | Modification made: Everyone stands in a circle holding hands. One person runs outside the circle (denner) and touches any other child's back, whom they want to challenge. The child who's back was touched runs and touches another child's back. | 9 | 10 | 10 | 10 | 0.8 | 0.9 | 0.9 | 0.9 | Maybe retained with the modification; No change needed; retain; retain; modification is fine; May have difficulty following the game; similar activity; | 8 | 3 | 0 | Accept |
| CO-ORDINATION |  |  |  |  |  |  |  |  |  |  |  |  |  |  |  |
| We move | C1 | The activity is an action song and will be demonstrated one step at a time, slowly by the therapist. With practice the speed maybe increased. Any modifications that the expert would like to suggest? | 10 | 9 | 9 | 9 | 0.9 | 0.8 | 0.8 | 0.8 | The therapist can demonstrate the actions in front of the child; Retain; No change; needs more simple command; Eliminate; Reduce the activity to two- three commands; Agree that activity is too complicated. | 4 | 6 | 1 | Accept |
| head, shoulders, knee | C2 | With demonstration, children maybe able to follow gradually after a few trials. Any modifications that the expert would like to suggest? | 11 | 11 | 11 | 11 | 1.0 | 1.0 | 1.0 | 1.0 | May be retained; Retain; Apt; can be demonstrated till they understand; retain; simplify the commands and retain; Agree with feedback, Eliminate either the cone or the body part | 8 | 3 | 0 | Accept |
| passing the parcel | C3 | This activity did not reach consensus only in the 'relevance' domain. Any modifications that the expert would like to suggest? | 6 | 7 | 8 | 9 | 0.5 | 0.6 | 0.7 | 0.8 | May be retained as the child needs coordination to pass the parcel to the next person's hand; The ball can be received by rotation of trunk from one side and passed on the other side. Retain; Appropriate; Eliminate; Does not require much coordination - eliminate; | 2 | 8 | 1 | Next Round |
| three legged race | C5 | Any modifications that the expert would like to suggest? | 7 | 6 | 7 | 5 | 0.6 | 0.5 | 0.6 | 0.5 | May be considered with safety precautions; Eliminate. Normal children also fall down in this item as very good co ordination is required between 2 individuals. Very risky; Appropriate; eliminate; Eliminate; Eliminate the activity , risk of fall is more; Eliminate because of safety; Concerned about safety - eliminate or modify - instead of tying legs - use hopscotch circles or a pathway to add difficulty; eliminate; safety? | 0 | 3 | 8 | Reject |
| hidden items | C6 | Any modifications that the expert would like to suggest? | 4 | 5 | 7 | 7 | 0.4 | 0.5 | 0.6 | 0.6 | Is irrelevant under coordination; Eliminate; Apt; retain; Eliminate; doesn't involve coordination component; Eliminate; Its a fun cognitive activity but would take time and does not fall under coordination -eliminate; | 0 | 1 | 10 | Reject |
| gongee | C7 | Any modifications that the expert would like to suggest? | 9 | 9 | 8 | 9 | 0.8 | 0.8 | 0.7 | 0.8 | May be retained under coordination; Retain. Can be put under Balance; No change; | 7 | 4 | 0 | Next Round |
| message receieved | C8 | Any modifications that the expert would like to suggest? | 4 | 5 | 6 | 6 | 0.4 | 0.5 | 0.5 | 0.5 | May be irrelevant if the child has sensory deficits; Eliminate; No change; eliminate; eliminate; Reatin the activity with modificaton. Standing in the circle, let them peroform hand and leg come cordination exercise; Agree with feedback, Eliminate; They might not understand the game. This doesnt fall into coordination domain. You could eliminate the activity. Option- every child gets a balloon that they need to keep pushing up with their hand and not let it touch the floor. If it falls to the floor, you are out. rather than pressing the hand, they can do tapping on the hand by either doing supination to pronation or pronation to supination; relevance? | 0 | 3 | 8 | Reject |
| BALANCE |  |  |  |  |  |  |  |  |  |  |  |  |  |  |  |
| crocodile can I cross the river | B2 | Modification: The denner is the therapist, who stands in one corner of the room. The children stand on the other side of the room facing the denner. They have to ask, "Crocodile, crocodile can I cross the river?".If the denner says 'yes, you can' then they walk 3 steps forward in tandem walking. If he says 'no, you cannot' then they have to walk back three steps in tandem walking. Please rate the modification. | 11 | 11 | 11 | 11 | 1.0 | 1.0 | 1.0 | 1.0 | Maybe retained with modification; Apt; retain; Retain; children will require supervision/assistance; Fun!; Complex commands | 10 | 1 | 0 | Accept |
| in the pond, on the bank | B3 | Children stand on a platform. The therapist commands 'in the pond' - children step down from the platform. The therapist commands 'on the bank'- children will step back on to the platform. | 11 | 11 | 11 | 10 | 1.0 | 1.0 | 1.0 | 0.9 | Maybe retained with modification; Apt; retain; Retain; children will require supervision/assistance; Fun! Complex commands | 8 | 3 | 0 | Accept |
| relay-hops and jumps | B7 | RELAY- WALKING Children are divided into teams and are made to stand in a straight line (one behind the other), on the opposite ends of the room. Then, they have to hold a ball over their head. And while counting one to fifty, they have to walk across the room and back to their team. Then the next person in line continues the same, till all children have completed a round. | 7 | 7 | 7 | 7 | 0.6 | 0.6 | 0.6 | 0.6 | May be retained if complexity is taken care of; Appropriate; retain; Insturctions are not clear. Provide the clear instruction; eliminate the counting part; Walking is too simple; Eliminate; | 1 | 4 | 6 | Reject |
| scavenger hunt | B8 | Any modifications that the expert would like to suggest? | 6 | 7 | 8 | 7 | 0.5 | 0.6 | 0.7 | 0.6 | Is not relevant under balance; Eliminate; No change; eliminate; eliminate; Not any object available in the surroundings, fix a object and ask the patient to walk tandem walk / sideways/ backwards; Agree with the feedbackEliminate; Eliminate; | 0 | 3 | 8 | Reject |
| hopscotch | B10 | Any modifications that the expert would like to suggest? | 9 | 9 | 10 | 9 | 0.8 | 0.8 | 0.9 | 0.8 | May be retained under balance; Retain; No change; Retain; Retain but eliminate jumping; Unsure; supervision | 8 | 3 | 0 | Accept |
| AGILITY |  |  |  |  |  |  |  |  |  |  |  |  |  |  |  |
| red light, green light | A5 | Any modifications that the expert would like to suggest? | 8 | 8 | 9 | 7 | 0.7 | 0.7 | 0.8 | 0.6 | May be retained for older children; Retain; Apt; instructions should be more clear; retain; instead of saying , show them red/ green sign boards; Agree with feedback, Use multiple cues like voice and picture, retain; Doesnt focus on agility much - can incorporate directional changes, zig zags etc, jump over obstacle | 5 | 6 | 0 | Next Round |
| prisoners | A6 | Any modifications that the expert would like to suggest? | 9 | 9 | 10 | 9 | 0.8 | 0.8 | 0.9 | 0.8 | May be retained; Retain; Apt; instruction should be simpler; retain; Agree with feedback, eliminate; Complex; complex | 7 | 3 | 1 | Accept |
| colour colour | A7 | Any modifications that the expert would like to suggest? | 10 | 10 | 10 | 10 | 0.9 | 0.9 | 0.9 | 0.9 | May be retained; Retain; Appropriate; retain; eliminate; retain; Eliminate the bubble-gum part; IQ combined? | 8 | 2 | 1 | Accept |
| queen of sheeba | A8 | Only feasibility domain did not reach consensus. Any modifications that the expert would like to suggest? | 9 | 9 | 9 | 10 | 0.8 | 0.8 | 0.8 | 0.9 | Maybe retained; Appropriate; eliminate; Activity is not having a aspect of agility; Retain; Not feasible as children may run around the entire place - difficult to control; commands should include activities of agility training | 8 | 2 | 1 | Shift to Balance & Accept |
| balloon up | A10 | Any modifications that the expert would like to suggest? | 9 | 9 | 9 | 9 | 0.8 | 0.8 | 0.8 | 0.8 | Safety concerns, May be retained; Appropriate; move to endurance; Retain; instructions need to be very specific; agree- safety concerns, Oh you have this activity already! this can be coordination! it can be with complex activity; supervision | 8 | 3 | 0 | Accept |
| ladders | A11 | Instead of jump, can we ask the children to cross over each child's leg to reach his spot? Any modifications that the expert would like to suggest? | 8 | 10 | 8 | 7 | 0.7 | 0.9 | 0.7 | 0.6 | May be eliminated because of safety concerns; Appropriate; retain; Retain with the modification; Better to adopt cross over; Retain with modification; do it with precaution they might jump at each other's legs- safety | 1 | 9 | 1 | Next Round |
| musical chairs with numbers | A13 | Any modifications that the expert would like to suggest? | 9 | 9 | 9 | 10 | 0.8 | 0.8 | 0.8 | 0.9 | Maybe retained; Retain; Apt; Retain; based on number activity included in agility , you can decide whether or not to include; Disagree with feedback, Retain; Choose agility tasks when numbers are called out - modify the activity | 9 | 2 | 0 | Accept |
| crows and cranes | A14 | Modification: Two teams will be formed, one will be called 'crows', and the other 'cranes'. The team members will make a line and stand side by side to each other. Both the teams will stand opposite to each other. When the therapist says crows, the crows have to run away towards the wall closest to them to be 'safe' while the cranes try to catch them before they reach the wall. Then they all come back to starting position. Next, if the therapist calls cranes they have to run and find a wall to be 'safe'. | 9 | 10 | 9 | 9 | 0.8 | 0.9 | 0.8 | 0.8 | The activity may be retained with clarity of instructions; Difficult to run and catch . Appropriate; retain; retain; Eliminate; Similar to a lot of the other games; complex commands | 8 | 3 | 1 | Accept |
| kho kho | A15 | Any modifications that the expert would like to suggest? | 6 | 6 | 6 | 5 | 0.5 | 0.5 | 0.5 | 0.5 | Maybe retained with safety precautions; Retain; No change needed; eliminate; eliminate; eliminate; too complex- Eliminate; Too complex - eliminate or modify; complex | 2 | 3 | 6 | Reject |
| FLEXIBILITY |  |  |  |  |  |  |  |  |  |  |  |  |  |  |  |
| mountain and valleys | F1 | Any modifications that the expert would like to suggest? | 9 | 10 | 10 | 9 | 0.8 | 0.9 | 0.9 | 0.8 | May be retained with precautions; retain; Apt; retain; retain; Agree with feedback, Use furniture instead of children to jump over or crawl under, Retain; safety | 7 | 4 | 0 | Accept |
| hula hoops | F2 | The investigators aim at generalized body flexibility. Any modifications that the expert would like to suggest? | 9 | 10 | 10 | 9 | 0.8 | 0.9 | 0.9 | 0.8 | Maybe retained; Children have laxity. precaution; Apt; retain; Retain; retain; General flexibility, Retain; safety | 8 | 3 | 0 | Accept |

| **DOMAINS / ITEMS** | **ACTIVITY** | **Modified Activity Description /**  **Query to Panel Experts** | **ROUND 3** | | | | | | | | | | | | |
| --- | --- | --- | --- | --- | --- | --- | --- | --- | --- | --- | --- | --- | --- | --- | --- |
|  |  |  | **Criteria** | | | | **CVI** | | | | **Expert Feedback / Comments / Suggestions** | **Reviewer Decision** | | | **Investigators Remarks** |
|  |  |  | Relevance | Appropriateness | Clarity of Instructions | Feasibility | Relevance | Appropriateness | Clarity of Instructions | Feasibility |  | Accept | Accept with modifications | Reject |  |
| STRENGTH |  |  |  |  |  |  |  |  |  |  |  |  |  |  |  |
| Duck Walk | S10 | Any modifications that the expert would like to suggest? | 10 | 9 | 10 | 10 | 0.9 | 0.8 | 0.9 | 0.9 | May be used for strength training; doable; Children with downs syndrome have a cognitive challenge as well,hence not sure about them able to perform this task. Probably very few with better cognition would be able to perform. In addition considerin hypermobility better to avoid this activity; it may be difficult for some children to understand or sustain the activity | 8 | 2 | 1 | Accept |
| ENDURANCE |  |  |  |  |  |  |  |  |  |  |  |  |  |  |  |
| cow and tiger | E8 | Can the activity be retained? Any modifications that the expert would like to suggest? | 10 | 10 | 10 | 10 | 0.9 | 0.9 | 0.9 | 0.9 | May be retained with safety precautions; can be retain; supervising is required; commands are still complex and only the children who are running have some physical activity; | 8 | 3 | 0 | Accept |
| CO-ORDINATION |  |  |  |  |  |  |  |  |  |  |  |  |  |  |  |
| passing the parcel | C3 | Can the activity be retained? Any modifications that the expert would like to suggest? | 10 | 10 | 10 | 11 | 0.9 | 0.9 | 0.9 | 1 | May be retained with the modification; doable; Second part of the activity takes away the co-ordination construct , instead of lounges or squats make them to perform co-ordination related acitivites and reduce the repetitions; Modification is relevant and doable; Still not sure if it falls under coordination. The activities chosen could me modified to include more coordination activities. | 6 | 5 | 0 | Accept |
| gongee | C7 | Any modifications that the expert would like to suggest? | 11 | 11 | 11 | 11 | 1 | 1 | 1 | 1 | May be shifted under balance domain with pegs; Doable; Include in balance activity; good for balance activity; supervised tandem walking | 4 | 7 | 0 | Shift to Balance & Accept |
| AGILITY |  |  |  |  |  |  |  |  |  |  |  |  |  |  |  |
| red light, green light | A5 | Any modifications that the expert would like to suggest? | 10 | 11 | 11 | 9 | 0.9 | 1.0 | 1.0 | 0.8 | May be retained; doable; Probably the children require some learning " they need learn red or green light" indicates what. Some amount of practice required for this activity; retain; Can be retained; Keep only forward movement. moving to right and left might cause lot of confusion. Also its not clear when the therapist will turn around. there should be some cue to indicate that the therapist will turn around. Command understood by all? Cognition level of all children | 7 | 4 | 0 | Accept |
| ladders | A11 | Any modifications that the expert would like to suggest? | 9 | 9 | 9 | 10 | 0.8 | 0.8 | 0.8 | 0.9 | May be retained for older children with the modification; doable; Just walking across doesn't come under agility comoponent. Eliminate the activity or include in balance or co-ordination; eliminate; Retain; this also could be included in balance components; | 3 | 7 | 1 | Shift to Balance & Accept |
